# Olfactory Information Transfer in Bumblebees and Its Variation According to the Food Resource

**DOI:** 10.3390/insects17090924

**Published:** 2026-09-03

**Authors:** Tomás Eloy González-Chinnici, Mateo Miró, Camila Belén Saldaña, Walter Marcelo Farina

**Affiliations:** 1 Laboratorio de Insectos Sociales, Departamento de Biodiversidad y Biología Experimental, Facultad de Ciencias Exactas y Naturales, Universidad de Buenos Aires, Buenos Aires 1428, Argentina; tomasgonzalezchinnici@gmail.com (T.E.G.-C.); miromateo08@gmail.com (M.M.); camilasaldana2802@gmail.com (C.B.S.); 2 Instituto de Fisiología, Biología Molecular y Neurociencias (IFIBYNE), CONICET-Universidad de Buenos Aires, Buenos Aires 1428, Argentina

**Keywords:** *Bombus pauloensis*, odor learning, foraging preferences, pollen, nectar

## Abstract

Within the framework of animal learning theory, information transfer refers to the generalization or transmission of acquired knowledge to novel situations, individuals, or environments. In pollinators, this process is critical for adaptive foraging and ecological success. Although olfactory information transfer has been demonstrated in bumblebees through classical conditioning or colony exposure, the influence of reward type (nectar vs. pollen) remains poorly understood. Here, we evaluated olfactory information transfer and decision-making in the South American bumblebee *Bombus pauloensis* using complementary laboratory and colony-based experiments. Under laboratory conditions, bees were either classically conditioned through the proboscis extension reflex (PER) paradigm using scented sucrose solution or exposed overnight to scented pollen and subsequently tested in a four-arm arena. In a more natural context, colonies were exposed to scented sucrose solution or scented pollen through in-nest feeders, after which foraging choices were assessed in a flight cage. Sucrose-conditioned bees exhibited an odor-contingent preference in the four-arm arena, consistent with associative learning under our conditions. In contrast, pollen exposure produced odor-associated trends. Linalool exhibited a bias, although airborne volatile concentrations were not directly quantified, representing a methodological limitation. Moreover, linalool-scented pollen induced intense feeder manipulation and increased mortality during overnight exposure, highlighting substantial physiological costs. Under colony conditions, 24-h exposure to scented pollen promoted significant odor-guided search preferences, particularly with linalool, whereas no significant effect of scented sucrose was detected under our 24 h in-nest exposure; individual feeder contact was not verified. Overall, behavioral responses varied jointly with odor identity and the combined operational framework; the salience of pollen-associated odors observed here is consistent with reward-specific effects but cannot be fully disentangled from differences in protocol structure between the sucrose and pollen experiments.

## Simple Summary

Bumblebees use smell to locate food and decide which flowers to visit. In this study, we examined how odors associated with pollen or sugar influenced the behavior of the South American bumblebee *Bombus pauloensis*. We found that these bees can learn an odor and later use it to guide their search for food, but the response depended on both the odor itself and the type of reward. Odors associated with pollen produced a clearer search response, especially linalool, when the association was acquired under captive conditions, whereas odors associated with sugar were also learned but had little effect in colony tests. We also found that exposure to pollen scented with linalool was associated with significantly higher mortality and signs of weakness in caged bees. These findings show that floral odors can strongly influence how bumblebees learn, share information, and choose where to forage, and they suggest that pollen-related odors may be particularly important in these decisions.

## 1. Introduction

To counteract the decline in natural pollination, many producers have turned to pollination services involving the use of commercial nests of non-*Apis* bees. However, this strategy should avoid the use of exotic pollinators [1,2]. In this context, it is crucial to develop pollination strategies that promote native pollinators. In this regard, a promising approach is the use of commercial nests of *Bombus pauloensis*, a bumblebee native to South America whose use as a commercial pollinator has increased in recent years, with successful applications in several crops [3,4,5].

In social insects, particularly honeybees (*Apis mellifera*), prior appetitive experiences inside the nest play a pivotal role in shaping foraging decisions in the field. For instance, in-hive exposure to scented sucrose solutions creates long-term olfactory memories that directly bias short-range foraging choices and patch selection outside the colony, even after extended retention intervals [6]. Similarly, honeybees can learn olfactory cues associated with pollen rewards, which subsequently guide flight orientation and reactivate foraging activity [7]. While these foundational studies demonstrate robust in-nest olfactory information transfer in honeybees, much less is known about how such social learning operates across different reward types (nectar vs. pollen) in bumblebees (*Bombus* spp.), whose distinct social structure, lack of strict division of labor, and resource storage dynamics may modulate how olfactory information is processed and retrieved.

The genus *Bombus* has been shown to exhibit a high level of cognitive development comparable to that of *Apis mellifera* [8,9]. In particular, the South American bumblebee *Bombus pauloensis* can learn odors under laboratory conditions using the proboscis extension response (PER) paradigm, demonstrating its ability to associate previously neutral odorants with rewards [9]. Moreover, this species forms stable long-term memories, enabling it to optimize foraging behavior [10,11]. These learning abilities, along with the fact that bumblebees present facultative endothermy, allowing them to forage in places and climates where honeybees cannot [12,13,14], make *B. pauloensis* a suitable candidate for use in pollination services through colony conditioning with odor-mimic-scented food to enhance the pollination of target crops, as has already been demonstrated in honeybees [15]. However, this application requires a deeper understanding of its cognitive abilities, particularly those involved in processing floral scent information.

Within the framework of learning theory, information transfer refers to the process by which knowledge acquired in one context is generalized or transmitted to new situations, individuals, or environments [16]. This process is essential for adaptation because it enables animals to use past experiences to solve novel challenges. Understanding how bees process and transfer floral-related information is therefore crucial for explaining their ecological success. Although olfactory information transfer has been demonstrated in bumblebees through classical conditioning or colony exposure [10,17], the influence of reward type (nectar or pollen) on this process remains poorly understood.

When foraging for pollen, bees learn floral features associated with its presence (in honeybees [7,18]; in bumblebees [19,20,21,22]). However, unlike nectar, the mechanisms through which pollen mediates learned floral associations remain unclear. While nectar provides the primary energy source for adult bees, pollen cannot be immediately metabolized for energy [23]. In bumblebees (such as *Bombus pauloensis*), the ecological dynamics of resource collection and colony utilization differ fundamentally from those of honeybees (*Apis mellifera*). Unlike honeybees, which exhibit rigid task specialization with distinct pollen, nectar, or water foragers, bumblebee workers lack strict task division and frequently collect both nectar and pollen simultaneously during a single foraging bout, often exploiting the same or different floral species [24]. Inside the colony, collected resources are deposited directly into designated wax pots for immediate or short-term communal consumption, without undergoing worker-to-worker trophallactic food transfer or extensive processing [17]. This distinct natural history implies that individual bumblebee foragers directly experience both reward types in close temporal proximity, which may directly modulate how olfactory cues associated with nectar versus pollen are learned, retained, and retrieved. These differences suggest that the cognitive flexibility of bumblebees across different types of rewards and foraging contexts may underlie their behavioral capacity to shape experience-based strategies and optimize foraging efficiency under fluctuating ecological conditions. This ability is especially relevant for insect pollinators because it may enhance species fitness by facilitating resource exploitation under changing environmental conditions [5,25,26].

This study aimed to evaluate the decision-making processes related to scented resource discrimination under various experimental conditions, ranging from captive conditions with free movement to natural conditions inside the nest and under unrestricted real-world final-use conditions. First, sugar is used as a reward during olfactory conditioning in the form of a sucrose solution, and the memory established will be tested in a four-arm choice device [27]. Subsequently, and with the objective of investigating whether this species can associate an olfactory cue with pollen as a primary, ecologically relevant reward, the same association will be assessed under operant conditions when experimental subjects are exposed overnight to that type of reward. Additionally, we will evaluate the association between an olfactory cue and a sugar solution or pollen but, this time, in more natural conditions by providing the olfactory cue with the reward inside the nest with the help of in-nest feeders and, subsequently, measuring if the bumblebees can recall the association in a flying cage in which they will have the opportunity to make a choice [10]. Based on these goals, it is hypothesized that olfactory conditioning with a sugar solution as a reward promotes an association that triggers an approach response towards the learned odor source during the recall phase, accompanied by an appetitive response such as PER. It is further hypothesized that exposure to scented pollen promotes an odor-reward association. Finally, it is suggested that the memory established can be evoked in a setting distinct from that of acquisition, depending on the reward offered.

## 2. Materials and Methods

### 2.1. Study Site, Animals, Behavioral Software, and Chemical Compounds

Experiments were performed at the Experimental Field of the Faculty of Exact and Natural Sciences of the University of Buenos Aires, Argentina (34°32′ S, 58°26′ W) during the spring–summer season of 2025–2026. A total of 36 nests of bumblebees (*Bombus pauloensis*) were provided by Brometan-Biobest Argentina S.A. (Burzaco, Prov. Buenos Aires, Argentina), which breeds this species in captivity. The colonies were housed in their original commercial boxes (27 cm × 24 cm × 20 cm) and were placed outdoors and allowed to forage freely for nectar and pollen from the flora available in the surrounding area.

Pure odors commonly present in floral fragrances [28,29], such as linalool (LIO), nonanal (NONA) and phenylacetaldehyde (PHE) (Sigma-Aldrich, Steinheim, Germany; linalool: purity 97%, CAS 78-70-6; nonanal: purity 95%, CAS 124-19-6; phenylacetaldehyde: purity 90%, CAS 122-78-1), were used during the experiments.

Methodological parameters—including odor concentration, delivery mode, and chemical identity—were tailored to the specific physical requirements of each experimental paradigm. In individual behavioral assays (e.g., PER and multi-arm olfactometers, Acrilium, Buenos Aires, Argentina), active airflow delivery was employed to generate precise, temporalized odor pulses without environmental contamination. Conversely, in-nest exposure relied on passive evaporation from filter paper matrices to maintain a steady, homogeneous olfactory environment within the confined nest volume without disrupting the colony microclimate through a constant forced-air draft. Odor concentrations were adjusted accordingly: lower effective liquid loads were used in enclosed exposure chambers to prevent volatile saturation and sensory adaptation, whereas higher concentrations were necessary in dynamic airflow setups to compensate for dilution in moving air streams. Finally, linalool (a monoterpene alcohol) and phenylacetaldehyde (an aromatic aldehyde) were chosen to represent distinct chemical classes commonly found in natural floral scent profiles [28], with an overnight incubation period for the pollen exposure and testing in the arena experiments and at the nest interface for the flight cage experiments.

In all experimental paradigms (both in-nest and individual assays), the odorized filter paper matrix was consistently placed in immediate physical proximity (<1 cm) to the food source (sucrose or pollen) to ensure precise co-localization of the olfactory cue and reward.

Behavioral assays throughout all the experimental series were recorded using BORIS software version 9.40.0 [30].

### 2.2. Experimental Series 1: Olfactory Information Transfer Using Sucrose Solution and Pollen as Rewards

**Figure 1:**
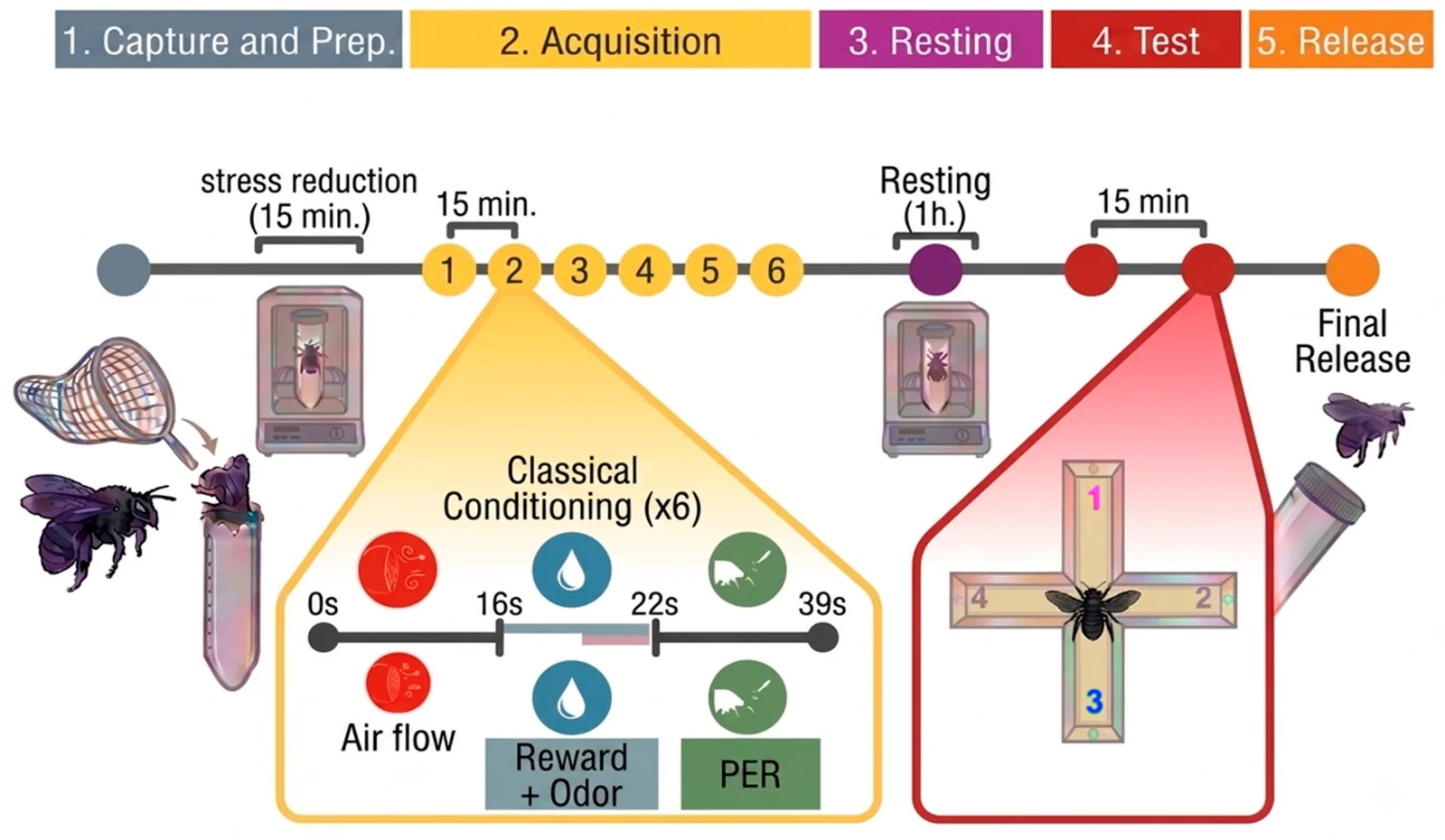
Schematic representation of the protocol described in experimental series 1 using sucrose solution as a reward. Capture and preparation (1): Bumblebees were captured with an entomological net, transferred to a tube and incubated for 15 min to ensure an unstressed physiological baseline in all bumblebees. Acquisition (2): A classical conditioning protocol to train bumblebees to a pure odor, comprising two experimental groups: LIO and NONA. The blue line indicates odor; the pink line indicates the sucrose solution reward. Resting (3): Experimental subjects were incubated for 1 h at 27 °C and 70% RH to reduce stress levels after the last FMPER event. Testing (4): Individuals were transferred to the four-arm arena to evaluate the response after the classical conditioning protocol, alternating between the LIO and NONA groups for each test. Release (5): After 15 min in the arena, bees were released (if the bumblebees were too exhausted to fly away, they were fed with a 50% *w*/*w* sucrose solution ad libitum until they flew away). Image created by the authors and refined using Gemini AI-assisted visualization tools (Google).

**Figure A1:**
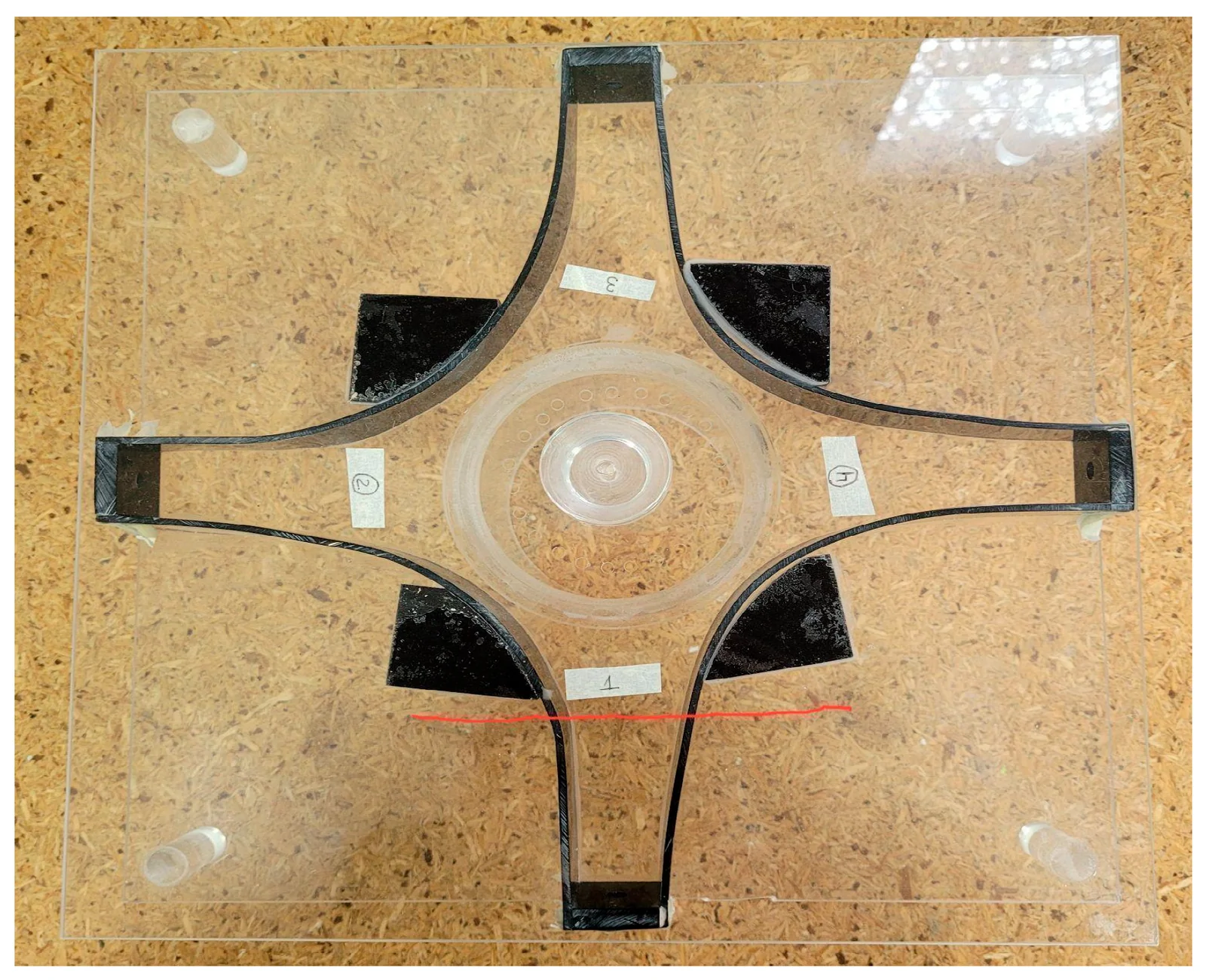
Four-arm arena showing the four quadrants from which the bumblebees could choose. The line where we considered quadrant choice is marked in red in quadrant 1 as an example. In each tip of the quadrants is where the syringes containing the filter paper with their respective odors were placed.

These behavioral assays aimed to evaluate information transfer in *Bombus pauloensis* bumblebees through odor-rewarded experiences using either sucrose solution or pollen as a reward. The assay began when free-flying bumblebees that had exited the hive were captured with an entomological net and carefully placed in 50 mL centrifuge tubes (Corning Inc., Corning, NY, USA). After being captured, bumblebees were kept individually in the centrifuge tubes, which were covered with mesh and placed in an incubator for 1 h at 27 °C and 70% relative humidity (RH). Either the olfactory conditioning procedure using sucrose solution as a reward (Section 2.2.1) or the scented pollen exposure overnight (Section 2.2.2) was conducted under captive conditions, allowing free movement for the experimental individuals inside the centrifuge tubes. After both experimental procedures, the behavioral evaluation was conducted in a four-arm experimental arena (Figure 1). While the four-arm arena is a validated device for measuring operant behavioral responses in *Apis mellifera* to test memory retention [27], there is no available information regarding its use with the genus *Bombus*. For this reason, we initiated this experiment to evaluate memory retention after sugar-odor conditioning (Figure A1).

#### 2.2.1. Olfactory PER Conditioning Using Sucrose Solution as a Reward to Validate a Four-Arm Arena

**Figure A2:**
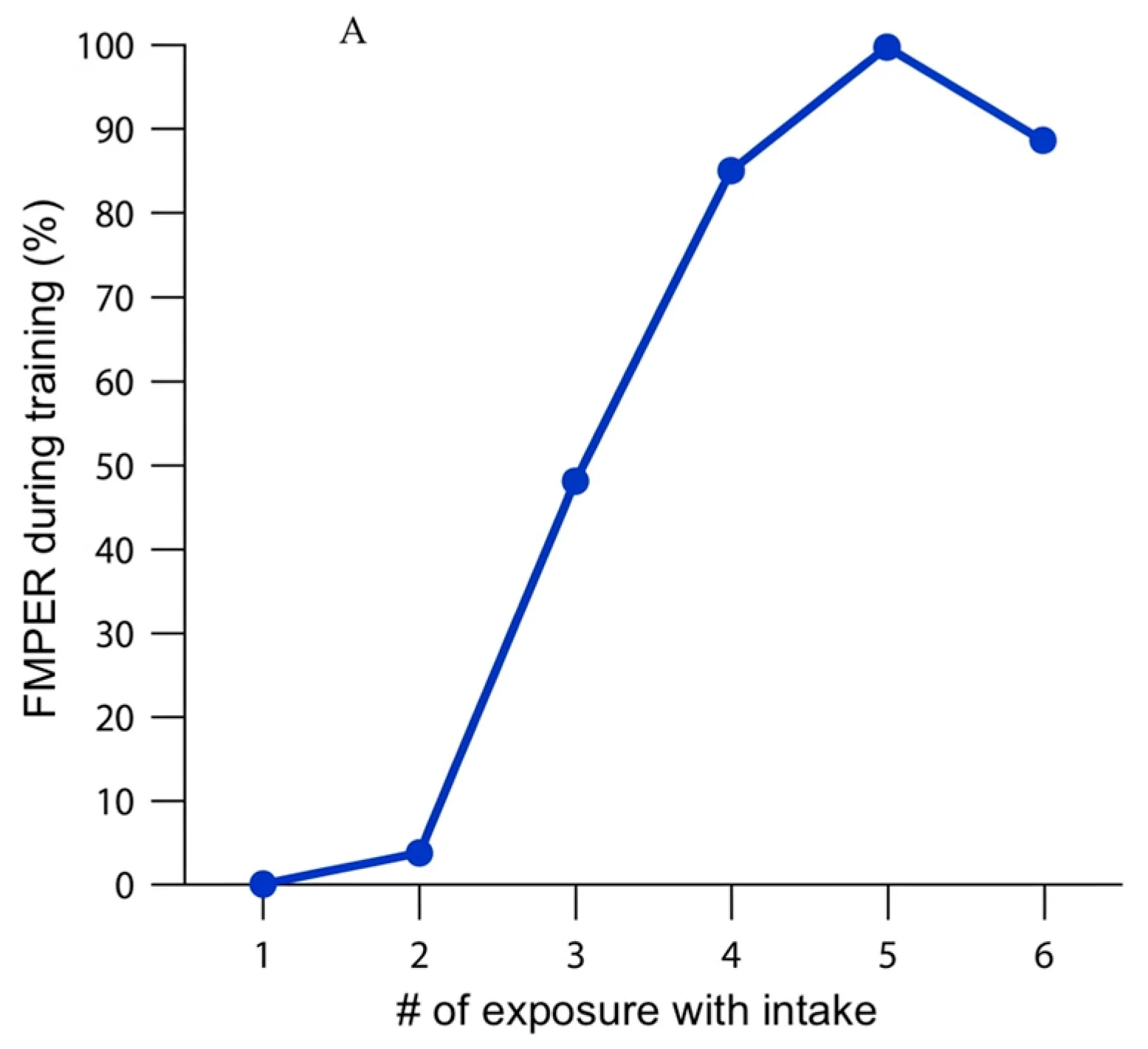

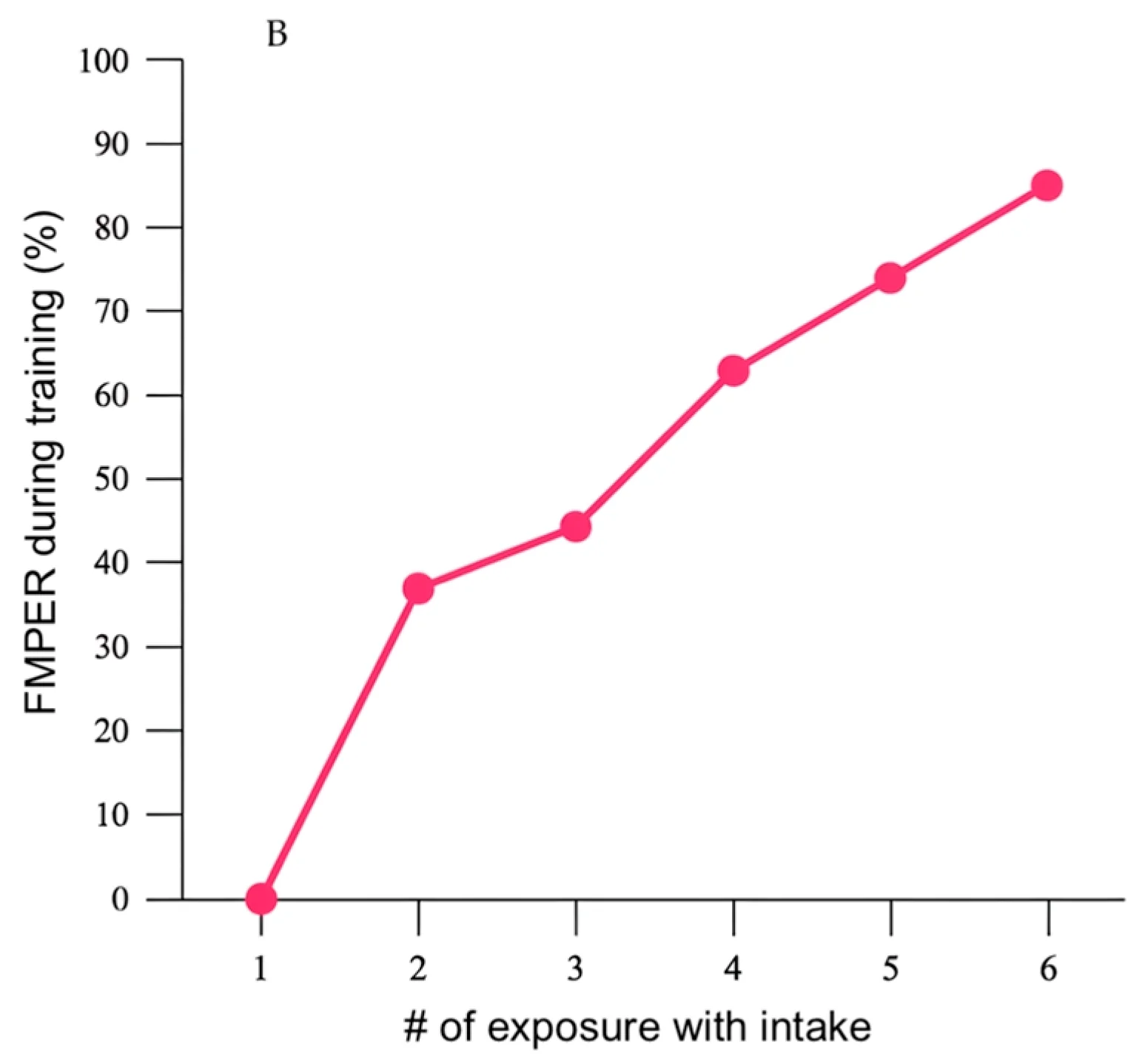
Acquisition responses (number of responses) during the training procedure. (**A**) Acquisition curve using nonanal as the conditioned stimulus (CS). (**B**) Acquisition curve using linalool as the CS. Treatment LIO, *n* = 30; treatment NONA, *n* = 27. For a detailed look into the behavior of bumblebees inside the arena, along with their proboscis extension after making a choice, check the following video: https://doi.org/10.5281/zenodo.21072769.

To validate our experimental device with which to test associative learning in bumblebees, we first performed classical conditioning using the proboscis extension response (PER) paradigm. The assay began by capturing bumblebees from 12 nests, as was explained above (Section 2.2). Individuals were transferred to 50 mL centrifuge tubes sealed with tulle mesh to ensure free movement and optimal ventilation. Subsequently, they were placed in an incubator at 27 °C and 70% RH for 15 min to allow stress levels to subside. These adaptations address the high susceptibility of this species to stress triggered by the harnessing techniques typically used for honeybees [31]. Consequently, the current protocol ensures confinement while allowing free movement—hereafter referred to as FMPER (free-moving proboscis extension reflex paradigm [11]). A representative diagram of the protocol is shown in Figure 1. This protocol remained identical across all cases. For conditioning, bumblebees were kept inside the mesh-covered 50 mL centrifuge tube. During the training, the conditioned stimulus (CS) was delivered via a continuous airflow (50 mL s^−1^; Keyrhon 3 w, Buenos Aires, Argentina) directed at the bumblebee’s head through the mesh. This secondary flow passed through a syringe containing a 0.2 cm × 1 cm filter paper soaked in 2 µL of pure odorant. Each of the six conditioning events lasted 39 s. The odor stimulus was presented for 6 s (6.25 mL s^−1^). After 3 s of exposure to the odor, they were offered 5 µL of 50% weight-by-weight (*w*/*w*) sucrose solution (henceforth: unconditioned stimulus, US). Two experimental groups were established: Group 1 (conditioned to linalool, LIO, as the CS) and Group 2 (conditioned to nonanal, NONA, as the CS). Bees that failed to respond to the sucrose solution by the final event of the training phase were excluded. Those that successfully associated the CS with the reward and formed associative memory were incubated at 27 °C and 70% RH for 1 h before the evaluation phase in the experimental arena (Figure A2).

Following the 1-h post-conditioning incubation period, bumblebees were individually transferred to the four-arm arena to evaluate their odor-guided responses. The arena consists of a central area where bumblebees enter through the top lid. At the bottom, an exhaust fan generates airflow, drawing scents from the four presentation quadrants. “Quadrant 1” contained a syringe loaded with a filter paper imbibed with 4 µL of LIO; “Quadrant 3” contained a filter paper with 4 µL of NONA; and “Quadrants 2 and 4” served as controls with no odor (“air”) [27]. The front of the arena was covered with white cardstock to minimize positive phototaxis bias toward windows. Between testing events, all contact surfaces were cleaned with 96% ethanol to eliminate pheromone traces. Spatial preference bias was mitigated by rotating the entire arena 90° clockwise for each subject. First choice inside the arena and proboscis extension were registered during the testing period (Figure 1).

#### 2.2.2. Choice Response After Overnight Scented Pollen Exposure 

**Figure 2:**
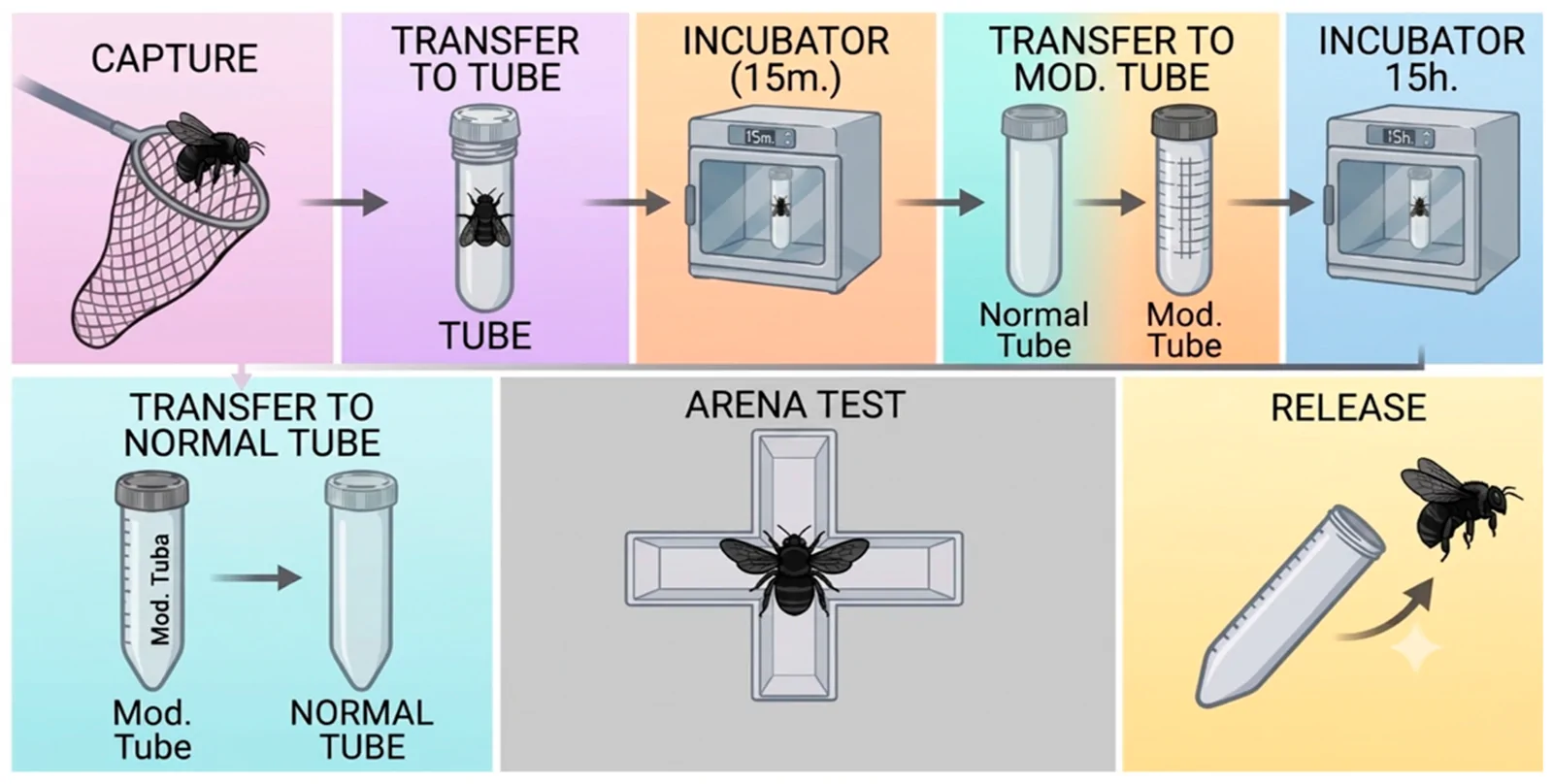
Schematic representation of the protocol described in experimental series 1 using pollen as a reward. Bumblebees were captured with an entomological net and transferred to centrifuge tubes. They were then incubated for 15 min to ensure an unstressed physiological baseline. Subsequently, bees were fed 50% *w*/*w* sucrose solution to satiety and transferred to modified centrifuge tubes containing the pollen reward in a pot, together with a filter paper carrying a pure odor stimulus. This is where individuals were assigned to the three treatment groups (control, LIO and NONA). Individuals were then incubated overnight for 15 h to ensure sufficient exposure to the scented pollen. Thereafter, bees were transferred to centrifuge tubes containing no aromatic stimuli. In these tubes, they were fed a drop (approximately 1 μL) of 50% *w*/*w* sucrose solution to provide enough energy for participation in the arena test. Bumblebees were then placed inside the four-arm arena to evaluate their response after pre-exposure to scented pollen, alternating randomly between the three treatments during each trial. After 15 min in the arena, bumblebees were released; if they were too exhausted to fly away, they were fed 50% *w*/*w* sucrose solution ad libitum until they flew away. Image created by the authors and refined using Gemini AI-assisted visualization tools (Google).

**Figure A3:**
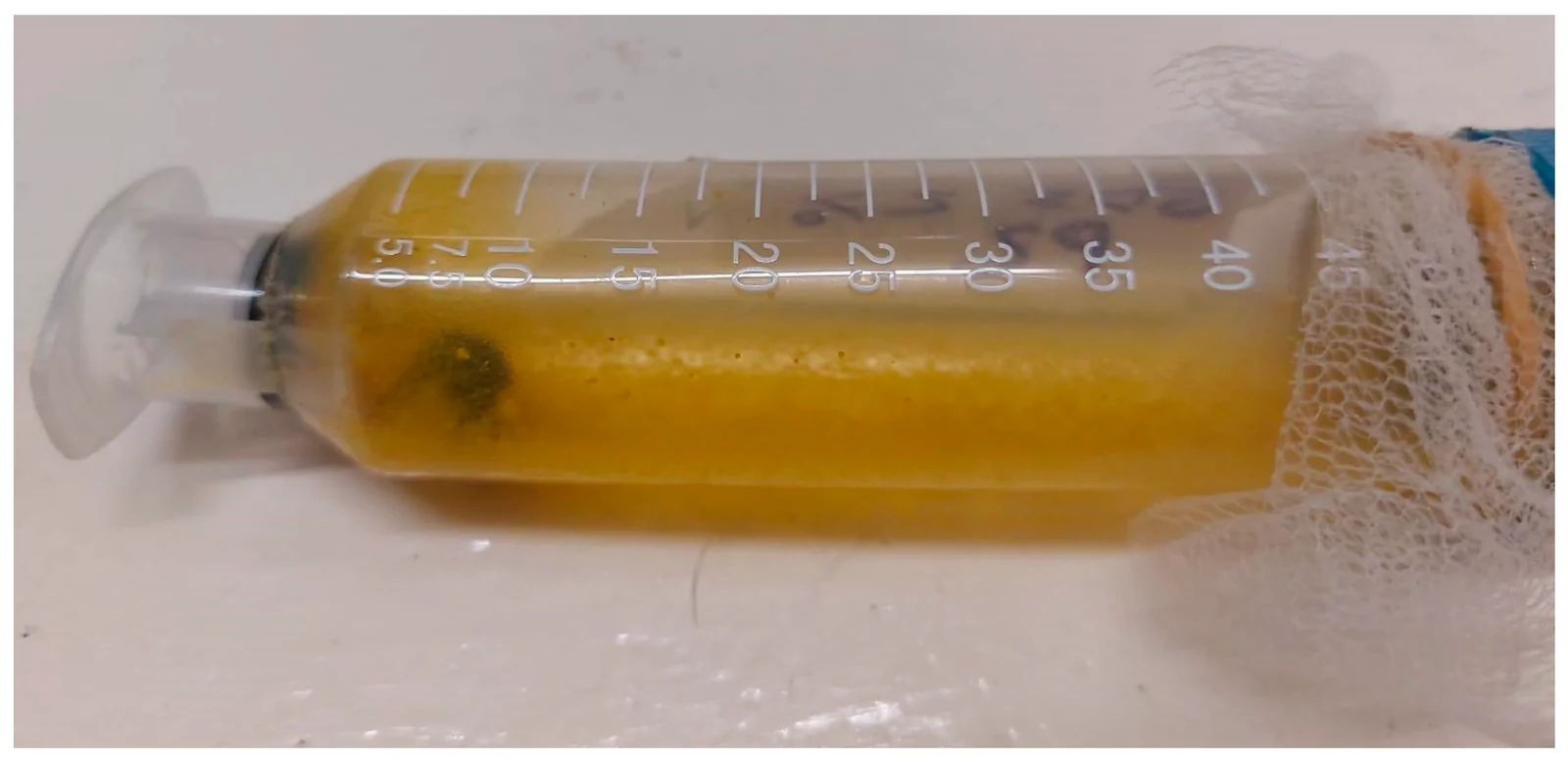
Modified tube and evidence of strong excitement during the overnight exposure, as seen by the spread of pollen along the complete length of the tube and the dead bumblebee right next to the pollen pot and LIO-scented filter paper.

We then evaluated whether the olfactory stimulus associated with pollen—a protein-and-lipid-rich resource that, unlike a sugar solution, cannot provide the primary energy source for adult bees—could effectively drive odor-guided responses in bumblebees. Bees were captured from 12 nests and incubated for 15 min at 27 °C and 70% RH to reduce stress levels. After incubation, they were fed to satiety with a 50% *w*/*w* sucrose solution. Satiated bumblebees were then transferred to modified 50 mL centrifuge tubes where the distal end was replaced with a syringe plunger acting as a “pot” to hold a pollen pellet (crushed multifloral pollen mixed with water; see Figure A3). A filter paper was placed at the junction and treated with one of these conditions: 2 µL LIO, 2 µL NONA, or left untreated (control). Subjects were incubated with the scented pollen for 15 h (from 16:30 to 07:30 the following day). Upon removal, interaction with the pollen (scratches or scattering) was verified in all cases. Bumblebees were then moved to standard centrifuge tubes, offered a small “incentive” drop of 50% *w*/*w* sucrose solution (without reaching satiety), and incubated for 2 h in a stimulus-free environment before being tested in the four-arm arena (Figure 2). Since the reward for this experimental series was pollen (a non-immediate food source) rather than sucrose (an immediate and appetitive short-term food source), variables measured included not only first choice and proboscis extension, as in the previous experimental series, but also latency to first choice and total time spent in each quadrant.

### 2.3. Experimental Series 2: Choice Response After In-Nest Exposure to Scented Sucrose Solution and Pollen

**Figure 3:**
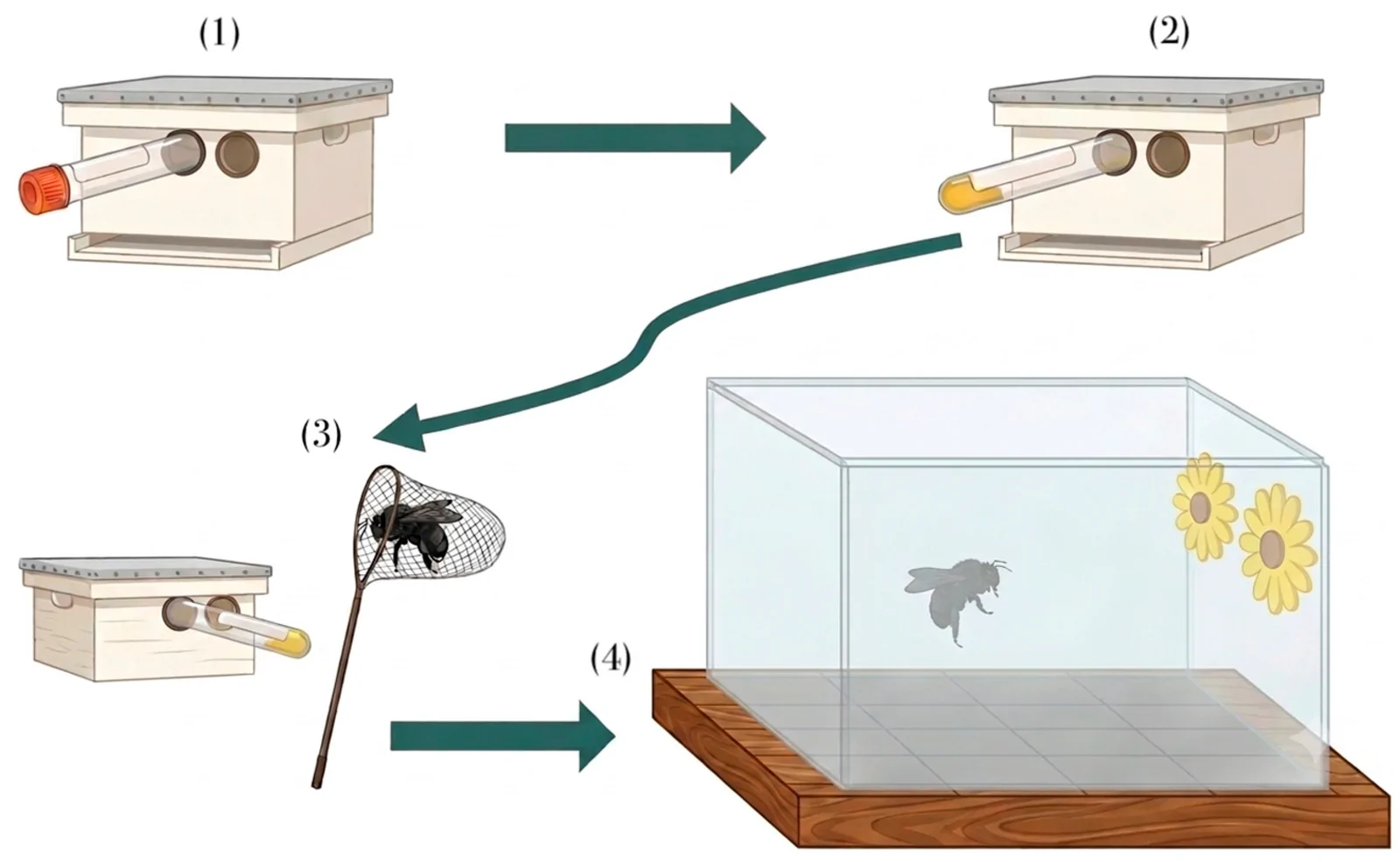
Experimental design for in-nest scented pollen pre-exposure and flight cage evaluation. (1) Pre-exposure with an unscented reward to familiarize bumblebees with the device; (2) 24 h pre-exposure with a scented appetitive stimulus (either sucrose or pollen, depending on the treatment); (3) capture on the following day; (4) immediate transfer to a flight cage featuring two yellow dummy flowers: one scented with the pre-exposure odor and one with a novel odor (bumblebees were captured in randomly assigned nest order and odor treatments in both sucrose and pollen trials). Image created by the authors and refined using Gemini AI-assisted visualization tools (Google).

**Figure A4:**
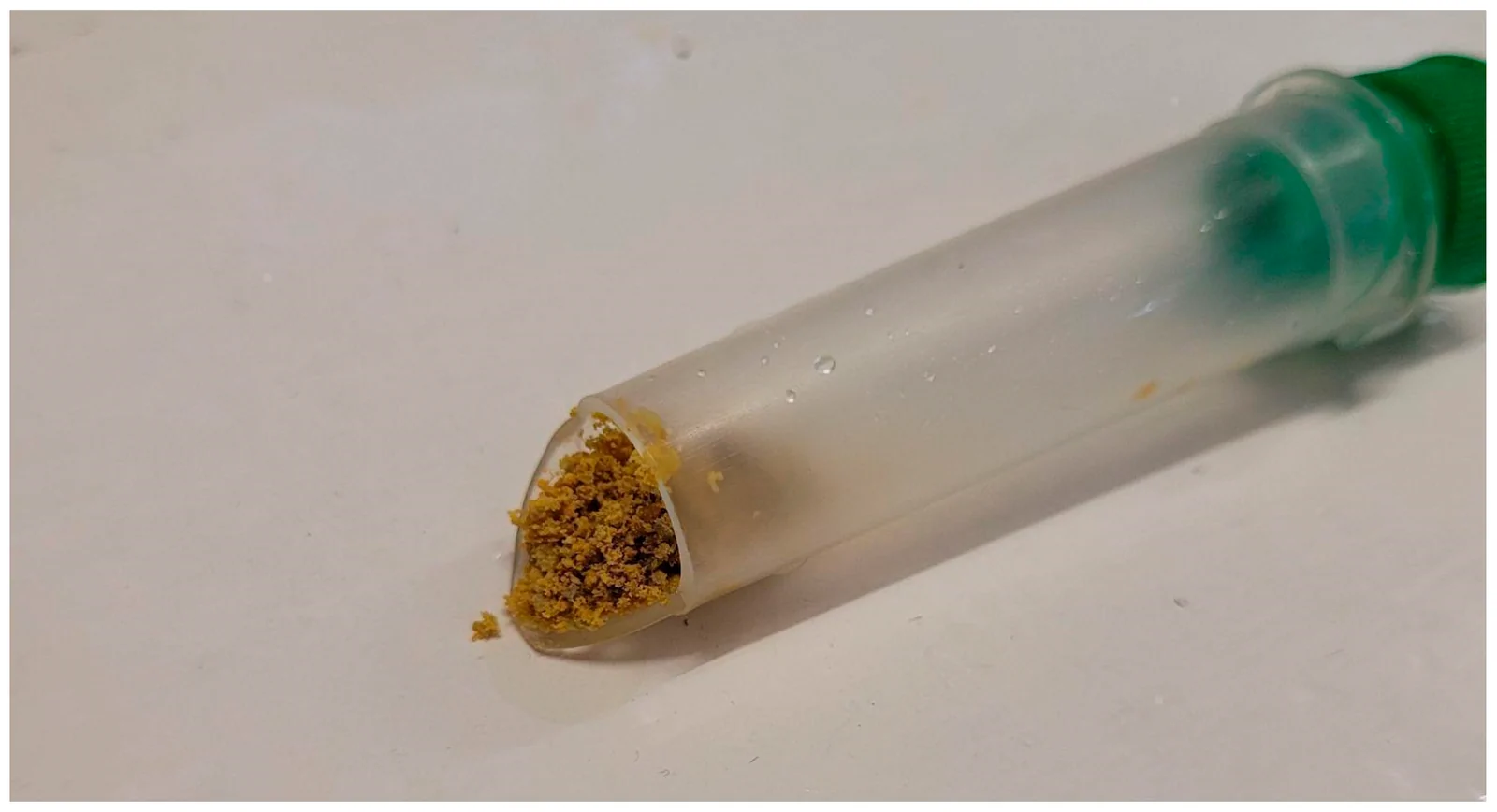
In-nest feeder loaded with pollen reward. The filter paper containing the CS goes underneath the pollen (see Figure A6).

**Figure A5:**
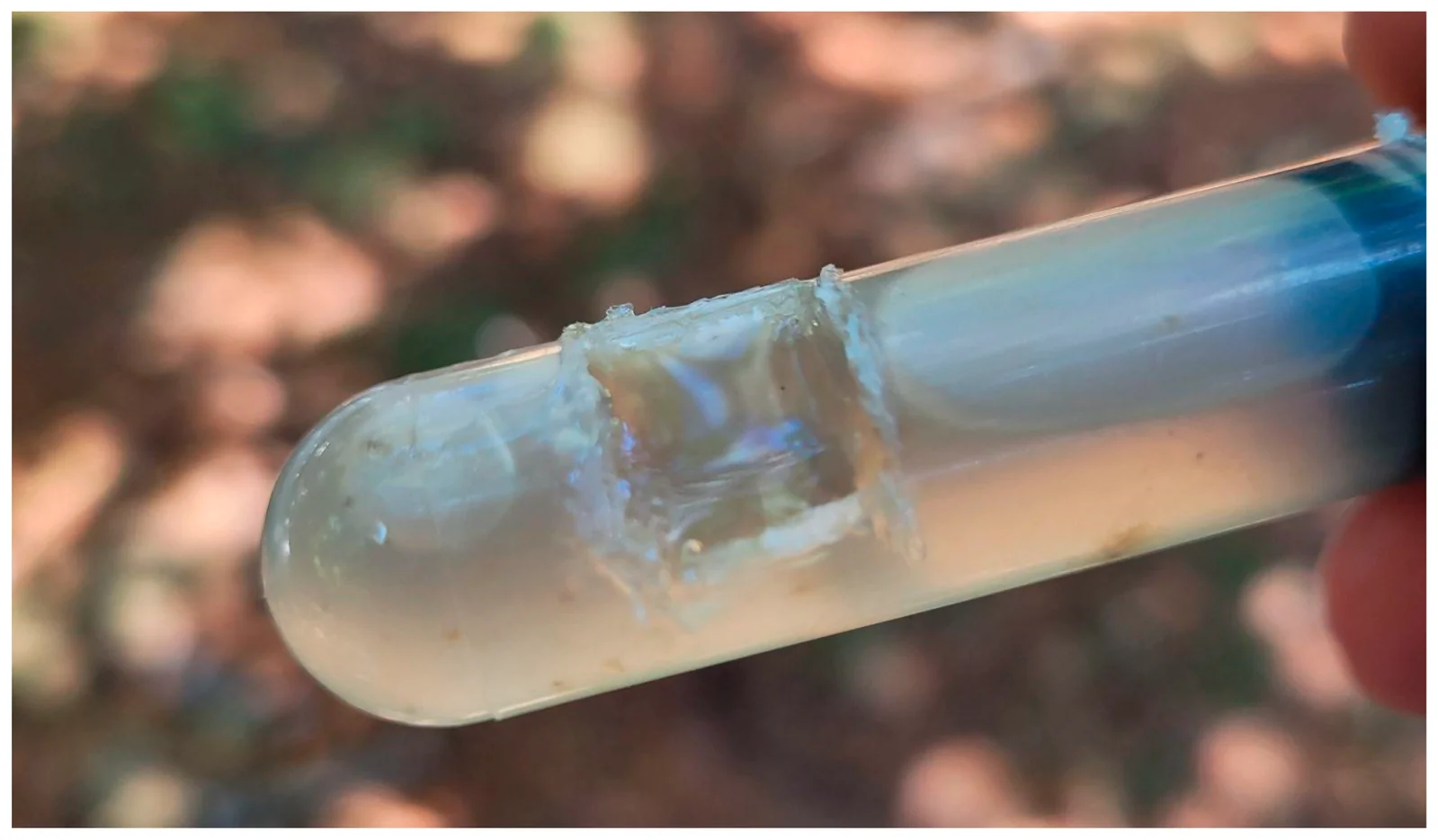
In-nest feeder loaded with sucrose reward. The filter paper containing the CS goes behind the opening for the solution (see Figure A6).

**Figure A6:**
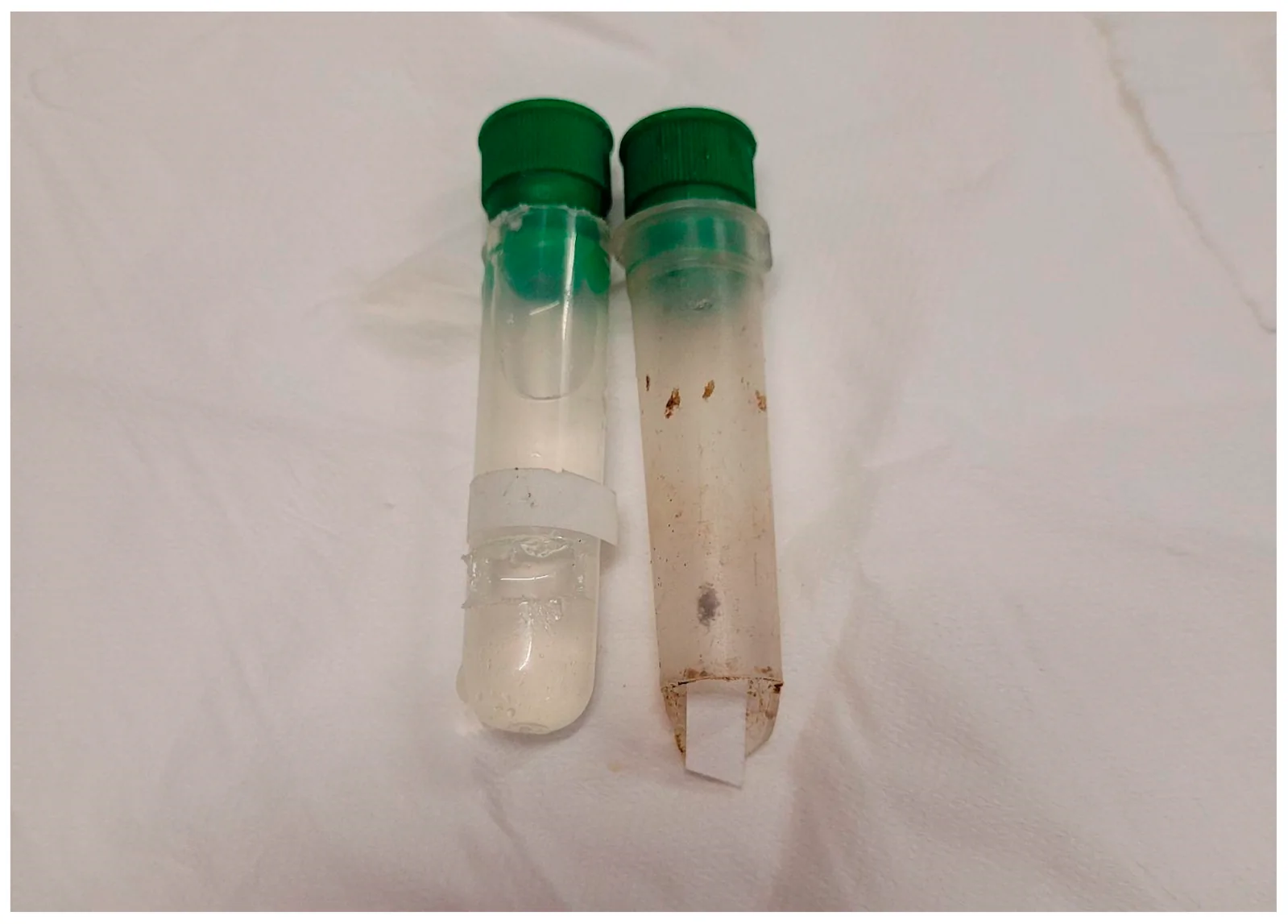
In-nest feeders with the filter paper attached. (Left) Sucrose reward feeder. (Right) Pollen reward feeders.

To evaluate responses under more natural, low-stress conditions, we used 12 nests for this experimental series. Plastic tubes filled either with 50% *w*/*w* sucrose solution or with crushed multifloral pollen were placed in one of the nests’ two entry/exit holes (Figure 3). For 6 days, bumblebees were familiarized with an unscented sucrose solution or pollen feeder. Once consistent consumption was confirmed, the tubes were replaced with (i) 50% *w*/*w* sucrose solution or (ii) pollen, both associated with a filter paper treated with either 2 μL of linalool (LIO) or phenylacetaldehyde (PHE). To evaluate whether odor-mediated information transfer generalized across broader structural classes rather than being restricted to a specific molecular pair, phenylacetaldehyde (an aromatic aldehyde commonly present in floral scent profiles [28]) was selected for flight-cage assays to complement initial paradigms utilizing nonanal (an aliphatic aldehyde) and linalool (a monoterpene alcohol). After 24 h of exposure, in-nest feeders were removed to check visitation and consumption of the reward. Thereafter, bumblebees were captured and transferred to an outdoor flight cage. Two yellow artificial flowers made with EVA foam were placed on the opposite side of the entrance of the flying cage, near the top at an equidistant distance from the borders of the cage. One of the flowers presented the pure synthetic odorant to which the bumblebees had been exposed (LIO or PHE), while the other flower presented a novel odor (LIO or PHE). Left and right for the correct odor were alternated, and the cage was rotated 90° after every bumblebee that was tested. A volume of 6 µL of each odor was impregnated on filter paper placed in the middle of each artificial flower. The flowers did not offer a reward. Flying cage experiments were carried out outdoors from the early morning until midday and then resumed in the afternoon (from 8 to 12 and from 13 to 15), with temperatures that ranged from 20 °C to 33 °C. Flying cage experiments were carried out outside since bumblebees are more likely to be active in high-luminosity areas in comparison to darker areas (personal observation of this species, [9]). Bumblebees were placed directly into the cage entrance and were given a maximum of 15 min to choose a flower. Searching time, time spent at each of the flowers, first choice and proboscis extension were the response variables considered. This experimental series allowed us to contrast the effects of a reward providing immediate appetitive reinforcement (50% *w*/*w* sucrose solution) against a non-immediate reward of primary importance for larvae and newly emerged adults (pollen) [23,32]. For details on the in-nest feeders used, see Figure A4, Figure A5 and Figure A6.

### 2.4. Statistical Analysis

All statistical analyses were performed in R v4.4.1 [33]. Behavioral data were analyzed using Generalized Linear Mixed-effects Models (GLMMs) to account for the hierarchical structure of the experimental design, including nest identity as a random effect in all models. Following this global assessment, the probability of choosing and choice directionality were analyzed using binomial logistic models with a logit link function. Choice latency and effective occupancy time for experimental series Section 2.2.2 were modeled using a Gamma distribution with a log link function to account for the non-normal distribution and heteroscedasticity of the data. Free-moving Proboscis Extension Response (FMPER) was analyzed using penalized logistic regression with Firth correction due to complete or quasi-complete separation in some experimental groups (logistf package [34]). Model simplification followed a stepwise approach, evaluating the significance of terms using Type III Wald chi-square tests (car package [35]). Estimated marginal means and a priori planned contrasts were performed to compare treatment levels against their respective controls using the emmeans package v. 2.0.4 [36]. For analyses involving more than two levels, pairwise comparisons were performed with Tukey adjustment to maintain the global significance threshold at alpha = 0.05. Model fit, overdispersion, and singularity were verified using the performance and see packages [37]. Data manipulation was conducted via dplyr v. 1.2.1 [38], and model outputs were tidied using broom.mixed v. 0.2.1.7 [39]. All visualizations were generated with ggplot2 v. 4.0.3 [40]. Foraging decisions within the flight cage, which included three behavioral outcomes (correct choice, incorrect choice, and no choice), as well as choice latency and effective occupancy time, were analyzed using a multinomial mixed-effects model with the mclogit v. 2.0-0 package [41], followed by Tukey-adjusted post hoc comparisons. For the flight cage and arena tests, analyses were restricted to individuals that successfully initiated a behavioral response within the allotted time. Nest identity was included as a random intercept in the modeling to account for colony-level non-independence. Other potential random factors, such as time of day and experimental day, were initially evaluated but dropped from the final models as they contributed negligible variance (near zero) and did not improve model fit (based on AIC comparisons), in alignment with previous findings [11].

## 3. Results

**Figure 4:**
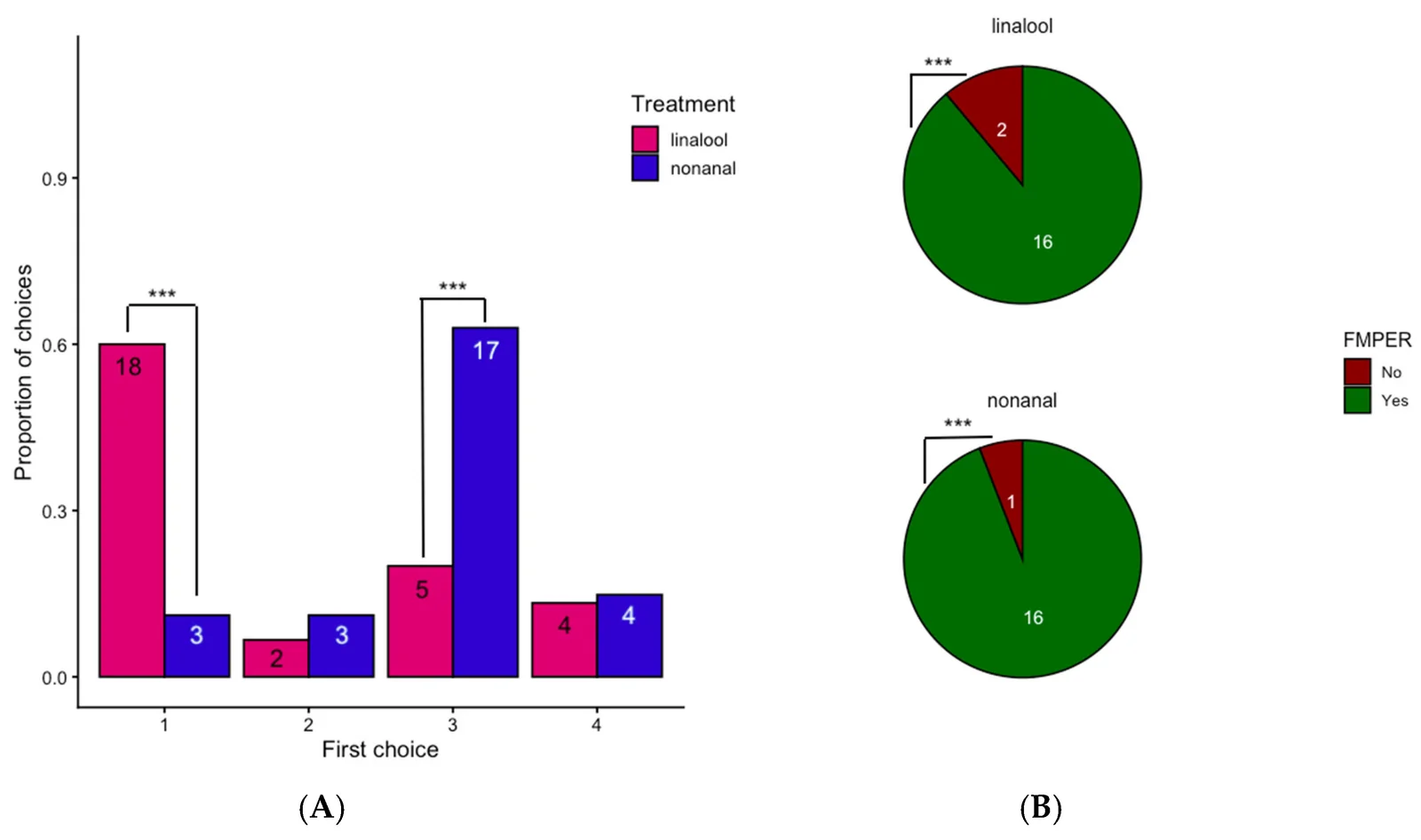
Arm choice and free-moving proboscis extension in bumblebees conditioned with sucrose solution. (**A**), proportion of arm choices; (**B**), FMPER responses shown as pie charts. Quadrant 1 contained linalool (LIO), Quadrant 3 contained nonanal (NONA), and Quadrants 2 and 4 contained no odor. Correct arm choice and proboscis extension within the chosen arm differed significantly between treatments. A binomial GLMM showed a significant treatment × quadrant interaction (Wald test: *z* = −2.49, *p* = 0.013). Post hoc contrasts confirmed an increased probability of choosing the quadrant containing the associated scent (Tukey test: Quadrant 1, LIO vs. NONA: *p* < 0.001, OR = 12; Quadrant 3, LIO vs. NONA: *p* < 0.001, OR = 0.15). For FMPER, Firth penalized logistic regression revealed a highly significant interaction between conditioning treatment and first quadrant choice (χ^2^ = 36.99, df = 7, *p* < 0.001). Proboscis extension was restricted to individuals whose spatial choice matched their conditioning treatment (16/18 LIO-conditioned bees in Quadrant 1 and 16/17 NONA-conditioned bees in Quadrant 3), confirming odor-specific memory recall in an operant context. *** *p* < 0.001. Treatment LIO, *n* = 30; treatment NONA, *n* = 27. Numbers inside boxes indicate the total number of bumblebees that chose the quadrant per treatment.

The choice of a specific quadrant was significantly influenced by the conditioned odor offered during the training phase (Figure 4). A clear preference for Quadrant 1 (linalool) was observed in individuals conditioned to associate a sugared reward with this scent. Similarly, individuals conditioned with nonanal as the CS showed a preference for Quadrant 3 (nonanal). Furthermore, a reward-seeking response was evident not only through the search for the aromatic source but also through the Free-Moving Proboscis Extension Response (FMPER) upon contact with it (Figure 4). To evaluate whether the choice probability depended on the conditioning treatment and odorant location, we fitted a Generalized Linear Mixed Model (GLMM) with a binomial distribution and logit link function. Fixed factors included treatment, quadrant, and their interaction, while individual identity nested within the colony was included as a random-effects structure (although it would prove to add no significant effect at all when running the model). The global analysis revealed a significant interaction between treatment and quadrant, driven by the distinct directional choices between the conditioning groups (Wald test: *z* = −2.49, *p* = 0.013). This significant global interaction supports the conclusion that the probability of choosing a target quadrant did not vary uniformly but was strongly dependent on the specific associative memory formed during conditioning. Post hoc directed contrasts confirmed a significant increase in the probability of choosing the quadrant containing the conditioned odor, consistent with differential learning. Specifically, significant differences were observed in quadrants containing the conditioned odor: Quadrant 1 (LIO vs. NONA): *p* < 0.001, OR = 12, 95% CI [2.95, 48.89], SE = 8.6, *z* = 3.5; and Quadrant 3 (LIO vs. NONA): *p* < 0.001, OR = 0.15, 95% CI [0.05, 0.49], SE = 0.09, *z* = −3.1. Additionally, the contrast between LIO and NONA treatments was significant (*p* < 0.001) for Quadrants 1 and 3, while contrasts for the remaining control quadrants were non-significant. Regarding the FMPER, a global penalized logistic regression (Firth’s method) was fitted to the data without artificial duplication, effectively accounting for the quasi-complete separation resulting from zero-response cells in mismatching and control conditions. The global model was highly significant (χ^2^ = 36.99, df = 7, *p* < 0.001, *n* = 57), revealing a robust interaction between conditioning treatment and chosen quadrant (e.g., treatment × quadrant interaction for NONA in Q3: β = 8.80, 95% CI [4.84, 14.93], *p* < 0.001; baseline PER response in Q1 for LIO: β = 1.89, 95% CI [0.72, 3.50], *p* < 0.001). This interaction was driven by a precise behavioral match: bumblebees almost exclusively extended their proboscis when their spatial choice coincided with their associative memory. Specifically, 16 out of 18 LIO-conditioned bees exhibited FMPER upon entering Quadrant 1, whereas none did so when entering Quadrant 3. Conversely, 16 out of 17 NONA-conditioned bees extended their proboscis in Quadrant 3, while none responded in Quadrant 1. In the air quadrants (2 and 4), FMPER was virtually absent across both treatments, ruling out generalized arousal or spurious reward-seeking behaviors. A video showing the behavior of a bumblebee in the arena after training can be seen in the link provided in Figure A2.

**Figure 5:**
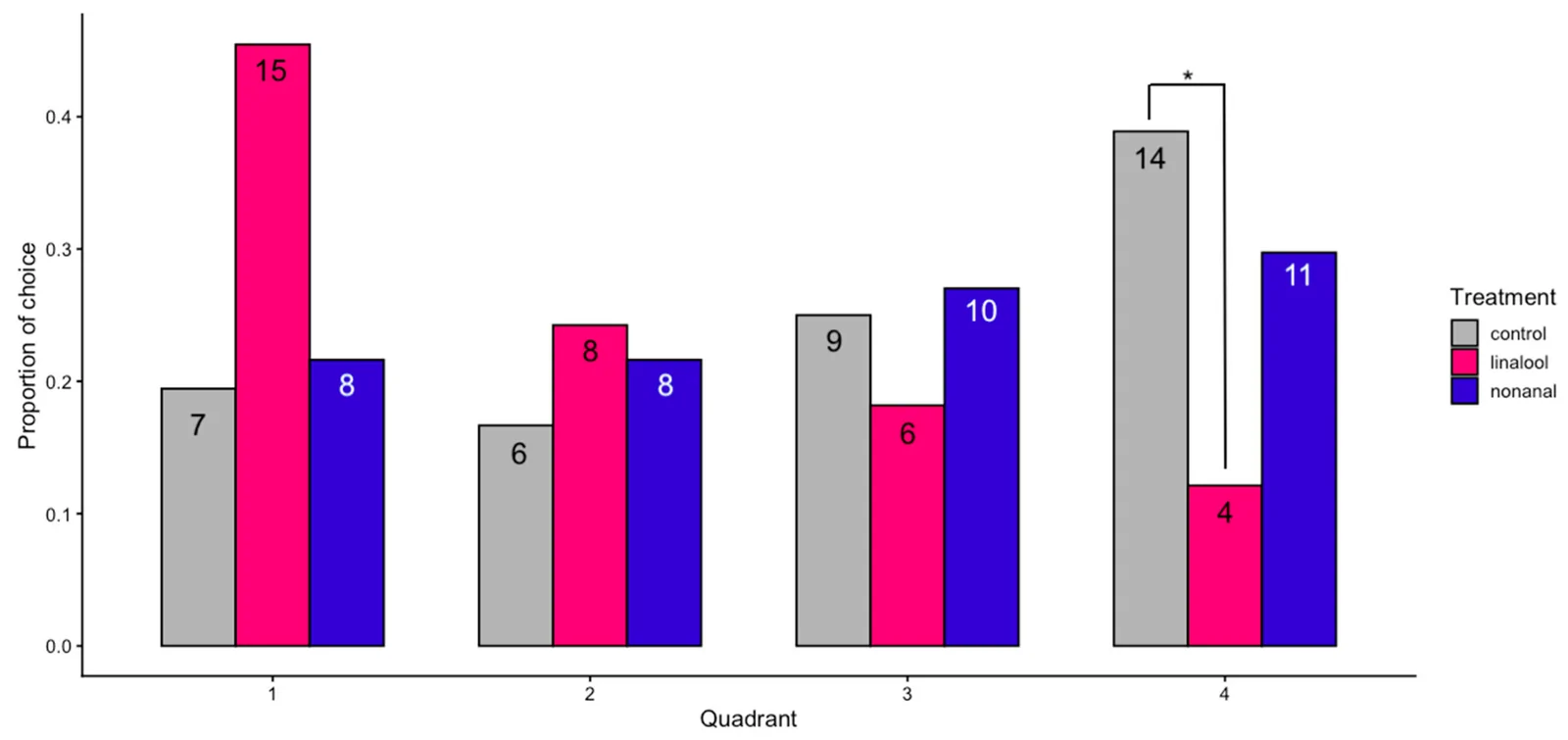
Proportion of quadrant choices after overnight exposure to scented pollen. Quadrant 1 contained linalool (LIO), Quadrant 3 contained nonanal (NONA), and Quadrants 2 and 4 contained no odor. Bees exposed to linalool-scented pollen showed a trend toward choosing Quadrant 1, the linalool-containing quadrant. However, this trend did not cross the significance threshold of *p* < 0.05. Bees in the nonanal treatment showed no directional trend. A significant contrast was detected in Quadrant 4, where control bees chose the air-only quadrant more often than linalool-exposed bees (Tukey test: control vs. LIO, *p* = 0.04, OR = 4.61, SE = 2.92, *z* = 2.41). Control, *n* = 36; LIO, *n* = 33; NONA, *n* = 37. No proboscis extension responses were observed. * *p* < 0.05. Numbers inside boxes indicate the total number of bumblebees that chose the quadrant per treatment.

The global GLMM analysis revealed a significant interaction between the scented-pollen overnight exposure and quadrant choice, marked by a strong effect of the linalool treatment within the target quadrants (Figure 5; Wald test: *z* = 3.31, *p* < 0.001). In contrast, post hoc comparisons within Quadrant 1 (LIO) did not meet the standard significance threshold (*p* < 0.05), exhibiting only marginal trends between control and linalool (*p* = 0.07, OR = 0.29, SE = 0.16, *z* = −2.26) and between linalool and nonanal (*p* = 0.07, OR = 3.02, SE = 1.6, *z* = 2.08). Conversely, a statistically significant difference was found in Quadrant 4 between control and linalool treatments (*p* = 0.04, OR = 4.61, SE = 2.92, *z* = 2.41). Just as in the previous experimental series (and the following ones after this), nest identity proved insignificant as a random effect. While these marginal trends in Quadrant 1 suggest a potential response pattern, they did not reach statistical significance and must be interpreted with caution.

**Figure 6:**
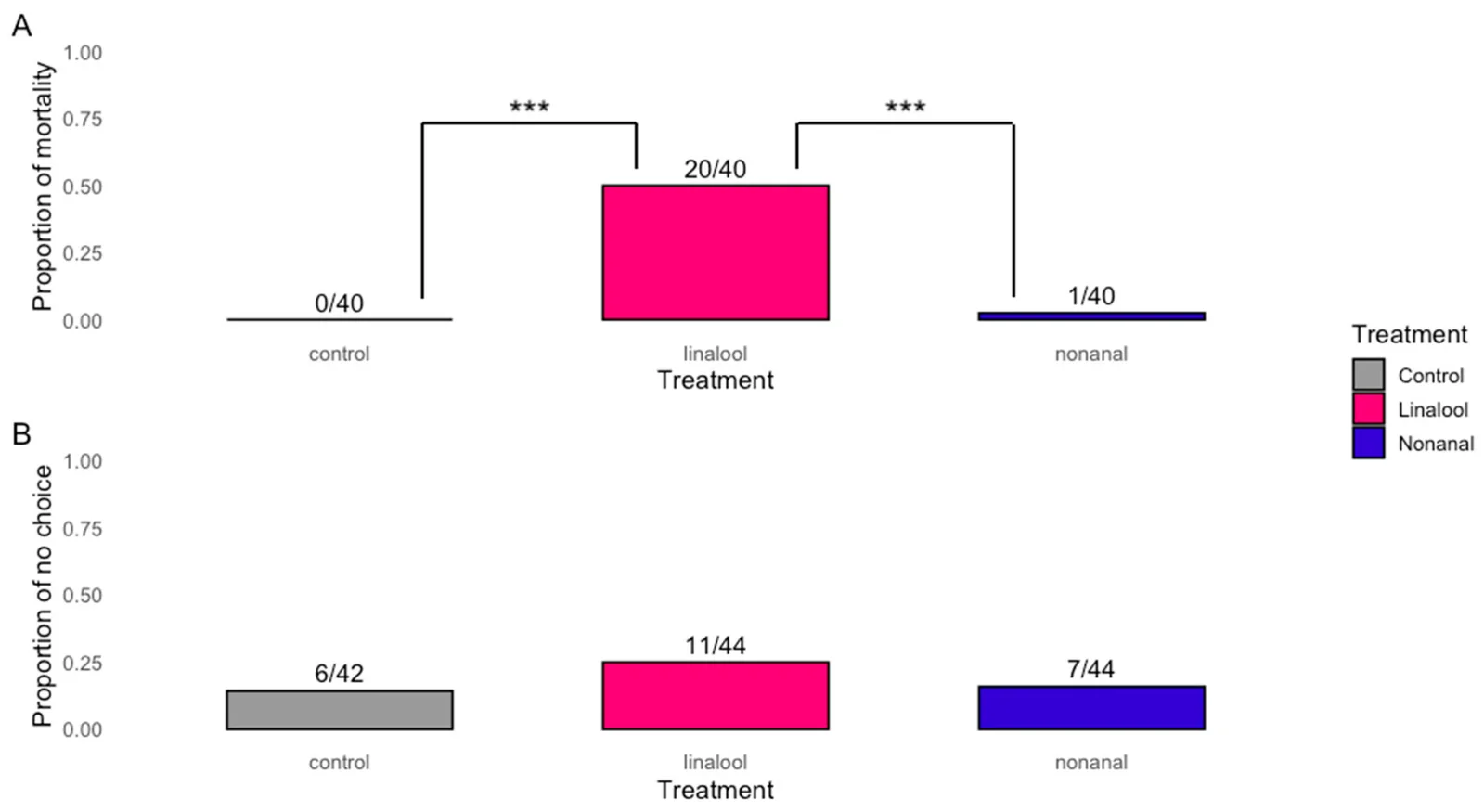
Mortality during overnight scented-pollen exposure and subsequent choice participation in the arena. (**A**), mortality rate (proportion) by treatment; (**B**), proportion of surviving individuals that did not choose an arm. Mortality differed significantly among treatments (overall Fisher’s exact test, *p* < 0.001). Pairwise post hoc comparisons with Bonferroni correction revealed significantly higher mortality in the linalool group compared to both the control group (*p* < 0.001) and the nonanal group (*p* < 0.001), whereas mortality did not differ between the control and nonanal groups (*p* = 1.000). In contrast, the probability of making a choice among surviving individuals did not differ significantly among treatments (GLMM, *p* > 0.05). *** *p* < 0.001.

The mortality measured after overnight exposure differed significantly between treatments. Due to structural zeros in the control group, Fisher’s Exact Test was performed, revealing highly significant differences (*p* = 2.39 × 10^−10^). Mortality was markedly higher in the linalool treatment compared to control and nonanal (Figure 6A), reaching 50% of the exposed individuals. Surviving individuals in the linalool group were visibly more lethargic, which likely affected their subsequent behavioral performance. A photo of the resulting overnight peak in activity with LIO treatment can be seen in Figure A3.

The probability of making a choice among surviving bees was analyzed using a binomial GLMM. The conditioning treatment had no significant effect on choice propensity (linalool vs. control: *z* = −1.23, *p* = 0.217; nonanal vs. control: *z* = −0.21, *p* = 0.834), suggesting that overnight scented pollen exposure did not modify the general disposition of surviving individuals to make a choice (Figure 6B).

**Figure 7:**
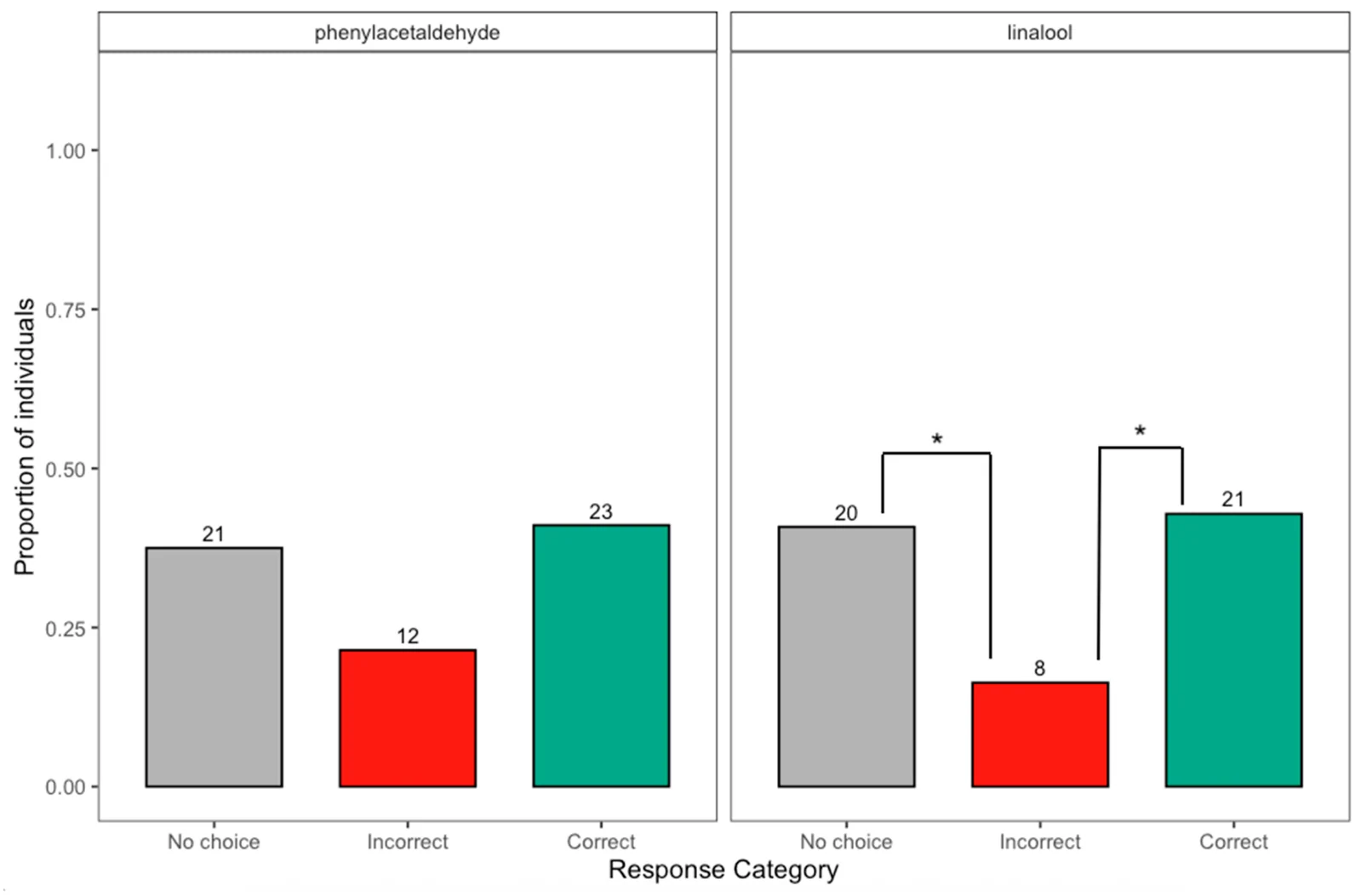
Distribution of foraging decision outcomes in the flight cage following in-nest scented pollen exposure. Bars show the frequency of correct choice, incorrect choice, and no choice responses after rewarded in-nest pre-exposure to phenylacetaldehyde (PHE; *n* = 56) or linalool (LIO; *n* = 49). Numbers above bars indicate absolute counts and percentages within each treatment. A multinomial mixed-effects model followed by Tukey-adjusted post hoc comparisons showed that, within the linalool group, bees were significantly more likely to make a correct than an incorrect choice (*p* = 0.027), whereas the phenylacetaldehyde group did not differ significantly from random accuracy (*p* = 0.13). Within the linalool group, no-choice responses were also more frequent than incorrect choices (*p* = 0.043). * *p* < 0.05.

**Figure 8:**
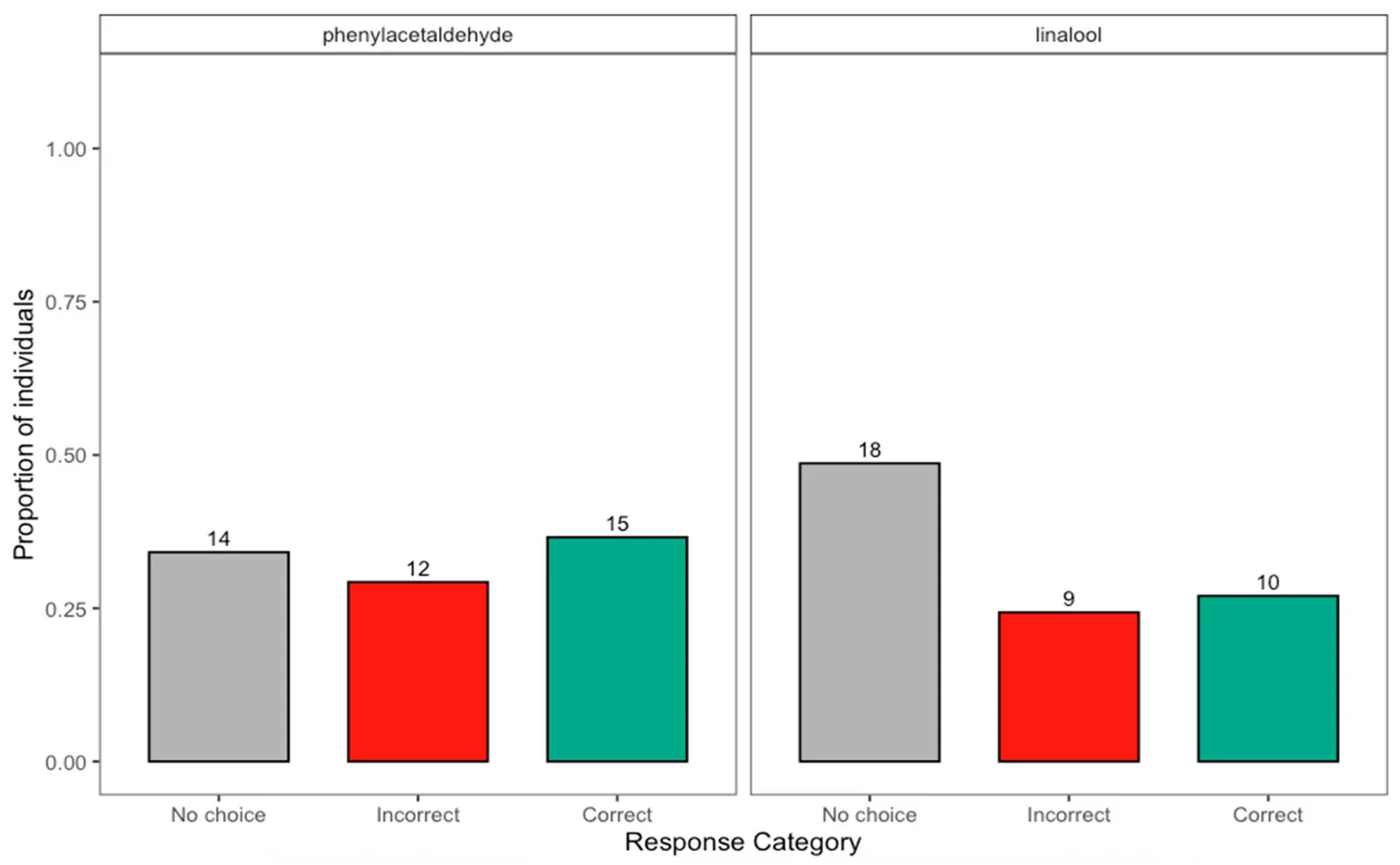
Distribution of foraging decision outcomes in the flight cage following scented in-nest sucrose-solution exposure. Bars show the frequency of correct choice, incorrect choice, and no choice responses in *B. pauloensis* individuals after rewarded in-nest pre-exposure to phenylacetaldehyde (PHE; *n* = 41) or linalool (LIO; *n* = 37) using sucrose solution as the reward. Numbers above bars indicate absolute counts and percentages within each treatment. A multinomial mixed-effects model followed by Tukey-adjusted post hoc comparisons revealed no significant differences between treatments or among behavioral categories within either treatment (all *p* > 0.58), indicating that sucrose-associated odor exposure did not significantly influence choice accuracy or general foraging motivation under these conditions.

The decision-making process within the flight cage was analyzed using a multinomial mixed-effects model to simultaneously evaluate the three possible behavioral outcomes: correct choice, incorrect choice, and no choice (Figure 7 and Figure 8). This approach allowed for a comprehensive assessment of foraging fidelity without the loss of information inherent in binary categorizations. Consistent with the reciprocal experimental design used in previous studies of in-nest learning, no non-stimulated control colony was utilized; instead, the use of two distinct odor treatments (linalool and phenylacetaldehyde) across independent colonies established a cross-over control [6]. This design reduces the likelihood that preference reflects innate attraction; however, without a naive baseline, inherent odor attractiveness cannot be excluded.

At the treatment level, the global model indicated that the overall distribution of behavioral categories did not significantly differ between the two odor pre-exposures. Specifically, the probability of making a correct choice over an incorrect one was statistically indistinguishable between the linalool and phenylacetaldehyde groups (β = 0.31 ± 0.55 SE; *z* = 0.57; *p* = 0.565). Similarly, the propensity to remain inactive (no choice) relative to making an incorrect choice did not vary between treatments (β = 0.36 ± 0.55 SE; *z* = 0.64; *p* = 0.519). Furthermore, the variance associated with the nest random effect was negligible (Var < 0.001), suggesting that these baseline decision patterns were highly consistent across the nine colonies sampled.

However, when examining the internal decision structure within each independent treatment via post hoc pairwise comparisons, a distinct behavioral pattern emerged solely in the linalool group. Individuals pre-exposed to linalool exhibited a clear directional preference, as the probability of making a correct choice was significantly higher than that of making an incorrect one (estimate = −0.26 ± 0.10 SE; *z* = −2.57; *p* = 0.027). Additionally, within this same treatment, the probability of a “no choice” outcome was also significantly greater than that of an incorrect choice (estimate = −0.24 ± 0.10 SE; *z* = −2.39; *p* = 0.043), whereas no significant difference was found between selecting the correct flower and remaining inactive (*p* = 0.987). In contrast, no internal differences among decision categories were observed within the phenylacetaldehyde group (all pairwise comparisons, *p* > 0.13). Delay in making a choice and time spent at each flower showed no significant differences.

In contrast to the patterns observed with scented pollen exposure, the olfactory treatment with a sucrose solution as a reward did not significantly influence any aspect of the behavioral response. No significant differences were found between treatments regarding the likelihood of making a correct choice over an incorrect one (β = −0.12 ± 0.60 SE; *z* = −0.20; *p* = 0.839) or in the propensity to remain inactive relative to making an incorrect response (β = 0.55 ± 0.59 SE; *z* = 0.94; *p* = 0.348). Notably, the variance associated with the nest random effect was considerably higher in this experimental series (Var. of no choice = 0.48; Var. of correct choice = 0.14) compared to the pollen trials, indicating a greater degree of behavioral heterogeneity between the two colonies utilized for this paradigm. Post hoc pairwise comparisons to test within treatments further confirmed the absence of a directional response within either group. For individuals pre-exposed to phenylacetaldehyde, probabilities were nearly identical across all outcomes, with no significant differences between correct and incorrect choices (*p* = 0.96) or between choosing and remaining inactive (*p* > 0.98). Similarly, in the linalool-exposed group, bumblebees performed at chance levels, showing no significant preference for the target flower over the incorrect one (*p* = 1.00) and no significant difference between making a choice and the “no choice” category (*p* > 0.58). Again, delay in making a choice and time spent at each flower showed no significant differences.

## 4. Discussion

Our results show that *Bombus pauloensis* can acquire and use olfactory cues associated with both nectar- and pollen-related rewards, but that the consequences of this learning depend on reward context and odor identity. Pollen-associated odors produced stronger odor-guided search responses than nectar-associated odors, with linalool eliciting the most robust effect after associative experience under captive conditions. In contrast, although sucrose-associated odors were learned, they had little influence on colony-level foraging responses. These findings suggest that pollen-related olfactory cues may play a particularly prominent role in guiding bumblebee resource exploitation. However, two limitations of our experimental setup should be considered from the outset. First, although fixed volumes of synthetic odorants were applied to feeders, airborne odor delivery and volatile concentrations inside the nest were not directly quantified, for example, through headspace chemical analysis. Second, prolonged overnight exposure to linalool-scented pollen resulted in increased mortality and signs of impaired condition in caged workers, pointing to potential physiological stress or toxicity at high exposure levels. Thus, while the observed behavioral responses confirm scent learning and reveal an interaction between odor identity and reward type, the dose-dependent nature of odor exposure and its associated survival costs should be considered when evaluating in-nest conditioning protocols.

### 4.1. Paradigm-Specific Expression of Sucrose Memory

A central finding of this study is the pronounced methodological contingency observed in sucrose-rewarded memory expression. While sucrose conditioning yields robust olfactory memory retention under standardized laboratory PER protocols, in-nest exposure to sucrose-scented reward failed to induce significant directional preferences in the flight-cage assay. This disparity highlights the profound influence of experimental framework and motivational state on cognitive expression. In the flight cage experiments, non-choosing behavior (“no choice”) was treated as an informative behavioral outcome rather than an experimental failure. Several non-mutually exclusive biological and methodological factors may explain why a bumblebee refrains from choosing a presented flower. First, individual foraging motivation and internal physiological state play key roles: Workers exiting the nest fully satiated may lack the drive to gather resources and thus remain uninfluenced by floral odor cues. Second, transferring individuals into the flight cage can induce a behavioral shift, redirecting worker activity from foraging toward exploratory, escape, or stress-induced behaviors in which floral stimuli lose salience. Finally, because rewards were provided at the colony level via in-nest feeders without tracking individual interactions, “no choice” responses may also reflect heterogeneity in feeder contact—specifically, individuals that never directly experienced the scented reward inside the nest. Although these mechanisms remain tentative and hypothetical due to the lack of individual tracking within the colony, incorporating non-selection as a distinct behavioral category captures the inherent variability of bumblebee decision-making under colony-level exposure.

In PER assays, individuals are typically food-deprived to maximize gustatory responsiveness, and memory is tested via an immobilized reflex under highly focused stimulus pairings. Conversely, during in-nest exposure, foragers consume sucrose ad libitum within a social colony setting, likely inducing temporary satiety that reduces subsequent foraging motivation in the flight cage. Furthermore, transitioning from an enclosed nest to a complex three-dimensional flight arena requires a cognitive shift from internal resource intake to active spatial navigation. Odor memories formed during passive or feeding inside the nest may not automatically translate into directional foraging decisions unless accompanied by relevant external foraging cues or a heightened energetic drive. Within this framework, bees experiencing the training scent in the nest atmosphere without directly contacting the scented sucrose feeder may exhibit no response to either familiar or unfamiliar scents, as prior reports confirm that frequent contacts with scented sucrose significantly increase the likelihood of subsequent responses [17,42].

### 4.2. Uniqueness and Mechanisms of Pollen-Associated Learning

A critical aspect when interpreting the comparative outcomes of this study is the inherent constraint on establishing a direct, perfectly symmetrical comparison of cognitive capabilities across reward types. Because nectar (sucrose) and pollen operate under fundamentally different energetic, physiological, foraging, and storage dynamics [24], adopting structurally divergent experimental paradigms was a biological necessity. While liquid food (sucrose) enables individual conditioning with immediate gustatory and metabolic feedback, pollen—due to its particulate nature and deferred processing within the nest—demands paradigms based on prolonged exposure within the colony context [7]. This methodological divergence, although ecologically justified, introduces a key epistemological limitation: observed performance differences between sucrose and pollen cannot be linearly attributed to an intrinsic cognitive advantage or associative hierarchy for one resource over the other. Instead, these behavioral variations also reflect disparities in learning and testing environments. The presence of a clear directional preference following exposure to scented pollen, compared to the exploratory response recorded after sucrose exposure, highlights how social colony dynamics and extended contact interact with resource identity. Physical and physiological constraints inherent to each resource define the boundaries of a unified cognitive baseline. Acknowledging these restrictions does not diminish the validity of our findings; rather, it underscores the complexity of cognitive ecology in pollinators, where maintaining ecological realism under controlled experimental conditions often requires sacrificing the metric symmetry of classical laboratory designs.

### 4.3. Modulatory Role of Odor Identity, Toxicity, and Future Directions

Behavioral responses were further modulated by the chemical identity of the olfactory stimulus. In the flight-cage experiment, the linalool group exhibited a significant correct-choice preference, demonstrating that this volatile promotes odor-guided responses and can successfully bias foraging decisions under semi-natural conditions. In this sense, previous work in honeybees has shown receptor-level sensitivity to linalool-related compounds [43], and bumblebee genomes have been shown to contain related olfactory receptor orthologs [44,45,46,47]. Although these comparative findings are compatible with the possibility that bumblebees are well equipped to detect common floral volatiles such as linalool, this mechanistic interpretation remains tentative and was not directly tested here.

In contrast, the phenylacetaldehyde group showed no significant directional bias. Because our experimental design did not include an unscented (no-odor) control group, it is not possible to definitively determine whether this lack of preference reflects a failure to learn the phenylacetaldehyde cue or if potential associative learning effects were canceled out by the innate valence (inherent attractiveness or repulsiveness) of the odor itself. Therefore, we constrain our conclusion to state that linalool is robustly learnable under these specific pollen-exposure conditions, whereas phenylacetaldehyde failed to show a similar effect.

Furthermore, an unexpected finding during overnight exposure assays was the elevated mortality rate (reaching ~50%) observed in the linalool-exposed group. While linalool is a widespread floral volatile [28,29], its high accumulation under enclosed nest conditions may exert complex physiological and behavioral costs. Several non-mutually exclusive mechanisms may account for this mortality: (i) Direct toxicity: One possible explanation is that prolonged exposure to linalool in the confined atmosphere of the experimental cages produced fumigant-like effects. Linalool is known to exert vapor-phase toxicity and to alter insect nervous system activity, including neuroexcitatory effects, raising the possibility that chronic exposure under restricted ventilation imposed physiological stress [48]. However, these effects have not been specifically tested in bees. (ii) Energetic depletion: Prolonged overnight exposure may induce continuous behavioral overstimulation and elevated metabolic rates, leading to energetic exhaustion if metabolic output surpasses intake. (iii) Pollinator filtering: From an ecological perspective, this behavior–toxicity coupling is consistent with the concept of floral volatiles acting as chemical filters, whereby secondary metabolites simultaneously attract effective pollinators while deterring florivores, nectar robbers, or other non-beneficial visitors [49,50].

Finally, while odor treatments were standardized by liquid volume and dilution following established behavioral protocols, airborne volatile release rates were not quantified via headspace solid-phase microextraction–gas chromatography (HS-SPME-GC). Because distinct volatile compounds exhibit different vapor pressures, potential variations in perceptual intensity could interact with odor identity. Future confirmatory assays integrating video-tracking of overnight activity, respirometry, dose–response trials in ventilated versus sealed chambers, and unscented controls might prove crucial to disentangling direct neurotoxicity from metabolic exhaustion and clarify the adaptive trade-offs of floral volatile exposure. Additionally, semi-natural flight-cage assays were conducted across an ambient temperature range (20–33 °C) reflective of natural foraging conditions for *Bombus pauloensis* during the spring–summer seasons. While thermal variation and time-of-day dynamics are known to modulate general flight activity and metabolic output in bumblebees, previous studies indicate that temperature effects on foraging are not significant within this optimal range, as bumblebees actively maintain their body temperature via facultative endothermy [12,13,14]. Furthermore, experimental sessions and treatments were strictly balanced across testing periods to prevent any potential systematic thermal confounds between groups.

## 5. Conclusions

The present results provide evidence that *Bombus pauloensis* can form associations between olfactory cues and reward context that are later expressed in operant settings such as the four-arm arena and the flight cage. Comparing sucrose, an immediate reward, with pollen, a non-immediate but ecologically relevant resource, under both captive and seminatural conditions, highlights that behavioral expression varies jointly with odor identity and the combined reward-paradigm context. These findings offer a useful framework for studying olfactory learning in bumblebees and for further examining the mechanisms underlying resource-specific effects.

## Data Availability

The raw data supporting the conclusions of this article are available through the following link: https://doi.org/10.5281/zenodo.21167565.

## References

[1] Garibaldi L.A., Carvalheiro L.G., Leonhardt S.D., Aizen M.A., Blaauw B.R., Isaacs R., Kuhlmann M., Kleijn D., Klein A.M., Kremen C. (2014). From research to action: Enhancing crop yield through wild pollinators. Front. Ecol. Environ..

[2] Aizen M.A., Aguiar S., Biesmeijer J.C., Garibaldi L.A., Inouye D.W., Jung C., Martins D.J., Medel R., Morales C.L., Ngo H. (2019). Global agricultural productivity is threatened by increasing pollinator dependence without a parallel increase in crop diversification. Glob. Change Biol..

[3] Riaño J.D., Pacateque E.J., Cure J.R., Rodríguez D. (2016). Comportamiento y eficiencia de polinización de *Bombus atratus* Franklin en pimentón (*Capsicum annum* L.) sembrado bajo invernadero. Rev. Colomb. De Cienc. Hortícolas.

[4] Cavigliasso P., Phifer C.C., Adams E.M., Flaspohler D., Gennari G.P., Licata J.A., Chacoff N.P. (2020). Spatio-temporal dynamics of landscape use by the bumblebee *Bombus pauloensis* (Hymenoptera: Apidae) and its relationship with pollen provisioning. PLoS ONE.

[5] Estravis-Barcala M.C., Palottini F., Macri I., Nery D., Farina W.M. (2021). Managed honeybees and South American bumblebees exhibit complementary foraging patterns in highbush blueberry. Sci. Rep..

[6] Arenas A., Fernández V.M., Farina W.M. (2007). Floral odor learning within the hive affects honeybees’ foraging decisions. Naturwissenschaften.

[7] Arenas A., Farina W.M. (2012). Learned olfactory cues affect pollen-foraging preferences in honeybees, *Apis mellifera*. Anim. Behav..

[8] Menzel R. (2001). Searching for the Memory Trace in a Mini-Brain, the Honeybee. Learn. Mem..

[9] Palottini F., Barcala M.C.E., Farina W.M. (2018). Odor Learning and Its Experience-Dependent Modulation in the South American Native Bumblebee *Bombus atratus* (Hymenoptera: Apidae). Front. Psychol..

[10] Nery D., Palottini F., Farina W.M. (2021). Classical olfactory conditioning promotes long-term memory and improves odor-cued flight orientation in the South American native bumblebee *Bombus pauloensis*. Curr. Zool..

[11] Pellettieri J., Farina W.M. (2026). Combined nectar nonsugar compounds enhance bumblebee cognition. Sci. Rep..

[12] Hines H.M. (2008). Historical Biogeography, Divergence Times, and Diversification Patterns of Bumble Bees (Hymenoptera: Apidae: *Bombus*). Syst. Biol..

[13] Cameron S.A., Hines H.M., Williams P.H. (2007). A comprehensive phylogeny of the bumble bees (*Bombus*): Bumble bee phylogeny. Biol. J. Linn. Soc..

[14] Lhomme P., Hines H.M. (2019). Ecology and Evolution of Cuckoo Bumble Bees. Ann. Entomol. Soc. Am..

[15] Farina W.M., Arenas A., Estravis-Barcala M.C., Palottini F. (2023). Targeted crop pollination by training honey bees: Advances and perspectives. Front. Bee Sci..

[16] Mackintosh N.J. (1994). Animal Learning and Cognition. Handbook of Perception and Cognition.

[17] Molet M., Chittka L., Raine N.E. (2009). How floral odours are learned inside the bumblebee (*Bombus terrestris*) nest. Naturwissenschaften.

[18] Grüter C., Arenas A., Farina W.M. (2008). Does pollen function as a reward for honeybees in associative learning?. Insectes Soc..

[19] Nicholls E., de Ibarra N.H. (2014). Bees associate colour cues with differences in pollen rewards. J. Exp. Biol..

[20] Muth F., Keasar T., Dornhaus A. (2015). Trading off short-term costs for long-term gains: How do bumblebees decide to learn morphologically complex flowers?. Anim. Behav..

[21] Muth F., Papaj D.R., Leonard A.S. (2016). Bees remember flowers for more than one reason: Pollen mediates associative learning. Anim. Behav..

[22] Russell A.L., Golden R.E., Leonard A.S., Papaj D.R. (2016). Bees learn preferences for plant species that offer only pollen as a reward. Behav. Ecol..

[23] Crailsheim K., Schneider L.H.W., Hrassnigg N., Bühlmann G., Brosch U., Gmeinbauer R., Schöffmann B. (1992). Pollen consumption and utilization in worker honeybees (*Apis mellifera carnica*): Dependence on individual age and function. J. Insect Physiol..

[24] Grüter C. (2026). Flower constancy in pollinators: A bouquet of agendas shapes interactions among mutualistic partners. Proc. R. Soc. B Biol. Sci..

[25] Russell A.L., Buchmann S.L., Papaj D.R. (2017). How a generalist bee achieves high efficiency of pollen collection on diverse floral resources. Behav. Ecol..

[26] Nery D., Palottini F., Farina W.M. (2024). The South American Black Bumblebee (*Bombus pauloensis*) as a Potential Pollinator of Alfalfa (*Medicago sativa*). Agriculture.

[27] Sandoz J.C., Laloi D., Odoux J.F., Pham-Delègue M.H. (2000). Olfactory information transfer in the honeybee: Compared efficiency of classical conditioning and early exposure. Anim. Behav..

[28] Knudsen J.T., Tollsten L., Bergström L.G. (1993). Floral scents—A checklist of volatile compounds isolated by head-space techniques. Phytochemistry.

[29] Raguso R.A., Pichersky E. (1999). New Perspectives in Pollination Biology: Floral Fragrances. A day in the life of a linalool molecule: Chemical communication in a plant-pollinator system. Part 1: Linalool biosynthesis in flowering plants. Plant Species Biol..

[30] Friard O., Gamba M. (2016). BORIS: A free, versatile open-source event-logging software for video/audio coding and live observations. Methods Ecol. Evol..

[31] Takeda K. (1961). Classical conditioned response in the honey bee. J. Insect Physiol..

[32] Teulier L., Weber J.M., Crevier J., Darveau C.A. (2016). Proline as a fuel for insect flight: Enhancing carbohydrate oxidation in hymenopterans. Proc. R. Soc. B Biol. Sci..

[33] R Core Team R: The R Project for Statistical Computing. https://www.r-project.org/.

[34] Heinze G., Schemper M. (2002). A solution to the problem of separation in logistic regression. Stat. Med..

[35] Fox J., Weisberg S., Price B. (2001). car: Companion to Applied Regression, Version 3.1-5. https://CRAN.R-project.org/package=car.

[36] Piaskowski J.L.R. (2026). emmeans: Estimated Marginal Means, Aka Least-Squares Means. R Package Version 2.0.3. https://rvlenth.github.io/emmeans/.

[37] Lüdecke D., Ben-Shachar M.S., Patil I., Waggoner P., Makowski D. (2021). Performance: An R Package for Assessment, Comparison and Testing of Statistical Models. J. Open Source Softw..

[38] Wickham H., François R., Henry L., Müller K., Vaughan D., Posit Software, PBC (2026). dplyr: A Grammar of Data Manipulation. https://cran.r-project.org/web/packages/dplyr/index.html.

[39] Bolker B., Robinson D., Menne D., Gabry J., Buerkner P., Hua C., Petry W., Wiley J., Kennedy P., Szöcs E. (2026). broom.mixed: Tidying Methods for Mixed Models. https://cran.r-project.org/web/packages/broom.mixed/index.html.

[40] Wickham H. (2016). ggplot2: Elegant Graphics for Data Analysis.

[41] Elff M. (2026). mclogit: Multinomial Logit Models for Categorical Responses and Discrete Choices. R Package Version 0.9.17.1. http://melff.github.io/mclogit/.

[42] McAulay M.K., Otis G.W., Gradish A.E. (2015). Honeypot visitation enables scent learning and heightens forager response in bumblebees (*Bombus impatiens*). Learn Motiv. Bee Insect Learn. Cogn..

[43] Claudianos C., Lim J., Young M., Yan S., Cristino A.S., Newcomb R.D., Gunasekaran N., Reinhard J. (2014). Odor memories regulate olfactory receptor expression in the sensory periphery. Eur. J. Neurosci..

[44] Karpe S.D., Jain R., Brockmann A., Sowdhamini R. (2016). Identification of Complete Repertoire of *Apis florea* Odorant Receptors Reveals Complex Orthologous Relationships with *Apis mellifera*. Genome. Biol. Evol..

[45] Karpe S.D., Dhingra S., Brockmann A., Sowdhamini R. (2017). Computational genome-wide survey of odorant receptors from two solitary bees *Dufourea novaeangliae* (Hymenoptera: Halictidae) and *Habropoda laboriosa* (Hymenoptera: Apidae). Sci. Rep..

[46] Mertes M., Carcaud J., Sandoz J.C. (2021). Olfactory coding in the antennal lobe of the bumble bee *Bombus terrestris*. Sci. Rep..

[47] Scheiner R., Page R.E., Erber J. (2004). Sucrose responsiveness and behavioral plasticity in honey bees (*Apis mellifera*). Apidologie.

[48] Gaire S., Scharf M.E., Gondhalekar A.D. (2019). Toxicity and neurophysiological impacts of plant essential oil components on bed bugs (Cimicidae: Hemiptera). Sci. Rep..

[49] Raguso R.A. (2008). Start making scents: The challenge of integrating chemistry into pollination ecology. Entomol. Exp. Appl..

[50] Raguso R.A. (2009). Floral scent in a whole-plant context: Moving beyond pollinator attraction. Funct. Ecol..

